# A Statistical Method for the Detection of Alternative Splicing Using RNA-Seq

**DOI:** 10.1371/journal.pone.0008529

**Published:** 2010-01-08

**Authors:** Liguo Wang, Yuanxin Xi, Jun Yu, Liping Dong, Laising Yen, Wei Li

**Affiliations:** 1 Division of Biostatistics, Dan L. Duncan Cancer Center, Baylor College of Medicine, Houston, Texas, United States of America; 2 Department of Molecular and Cellular Biology, Baylor College of Medicine, Houston, Texas, United States of America; 3 Department of Pathology, Baylor College of Medicine, Houston, Texas, United States of America; 4 Beijing Genomics Institute, Chinese Academy of Sciences, Beijing, China; Centre de Regulació Genòmica, Spain

## Abstract

Deep sequencing of transcriptome (RNA-seq) provides unprecedented opportunity to interrogate plausible mRNA splicing patterns by mapping RNA-seq reads to exon junctions (thereafter junction reads). In most previous studies, exon junctions were detected by using the quantitative information of junction reads. The quantitative criterion (e.g. minimum of two junction reads), although is straightforward and widely used, usually results in high false positive and false negative rates, owning to the complexity of transcriptome. Here, we introduced a new metric, namely Minimal Match on Either Side of exon junction (MMES), to measure the quality of each junction read, and subsequently implemented an empirical statistical model to detect exon junctions. When applied to a large dataset (>200M reads) consisting of mouse brain, liver and muscle mRNA sequences, and using independent transcripts databases as positive control, our method was proved to be considerably more accurate than previous ones, especially for detecting junctions originated from low-abundance transcripts. Our results were also confirmed by real time RT-PCR assay. The MMES metric can be used either in this empirical statistical model or in other more sophisticated classifiers, such as logistic regression.

## Introduction

Alternative splicing (AS), which invalidates the old theory of “one gene one protein”, enables higher eukaryote to produce large number of transcripts with limited number of genes, and has been proposed as a primary driver of the evolution of phenotypic complexity in mammals [1]. In human, ∼95% of multi-exon genes undergo alternative splicing, which explains the numerical disparity between the low number of human protein-coding genes (∼26,000) and the high number of human proteins (more than 90,000) [2], [3]. Alternative pre-messenger RNA splicing also influences development, physiology, and disease; many studies have reported the existence of cancer-specific alternative splicing in the absence of genomic mutations (for a review see [4]).

Several methods have been applied to detect AS events. Expression Sequence Tag (EST) was the first widely used technology and played a leading role in detecting AS events. However, except for the relatively high cost, EST technology has many other limitations including genomic contamination, cloning bias, paralog confusing, 3′ gene bias and low sensitivity in detecting low abundance transcripts. Besides, it also requires great efforts for data interpretation [5]. Microarray technologies have also played a prominent role in shaping our understanding of the complexity of transcriptome [1], [6], [7]. Recently, whole-transcript microarrays were used to monitor 24,426 alternative splicing events in 48 human tissues and cell lines [8]. Although this technology has been used extensively, limitations still persist; including limited probe coverage, cross-hybridization artifacts, requirement of previously known gene structures and difficulties in data analysis, etc.

More recently, rapid progress in the development of massively parallel sequencing such as Illumina/Solexa or Applied Biosystems/SOLiD, has provided people unprecedented opportunities to interrogate plausible alternative RNA splicing. Using these technologies, tens of millions of short tags (25–75 bases) can now be simultaneously sequenced at less than 1% the cost of traditional Sanger methods. Deep sequencing of transcriptome (RNA-seq) quickly becomes the most powerful technique to interrogate the whole transcriptional landscape [9], including both known transcript quantification and novel transcript discovery. Theoretically, all splicing events as well as chimeric transcripts can be directly detected [10]. However, the RNA-seq downstream data analysis still remains a big challenge.

Several major alternative splicing forms, such as exon skipping, mutually exclusive exon, alternative first/last exon and intron retention, can be detected by simply mapping RNA-seq reads to hypothetical splicing junctions. The reliability of a splicing junction is determined by: 1) number of reads mapping to the junction (junction reads); 2) number of mismatches on each mapped read; 3) read mapping position on the junction, i.e. how close is the center of the read to the junction itself. The shorter the distance is, the less likely that this mapping is simply by chance; 4) Mismatch position on junction read, e.g. mismatches occurring at both ends of reads are more likely due to the sequencing error, while those occurring in the middle of read are more likely to be polymorphisms [11].

However, most previous studies only considered the first quantitative information of junction reads, i.e. an exon junction is considered to be real if it has more than *R* junction reads (*R* = 1 or 2) [12], [13]. This read-counting method, as demonstrated in the results, has both high false positive and false negative rates. On the other hand, in one of the two earliest pioneering human transcriptome studies, Pan *et al* [3] used features similar to those described above to train both linear and nonlinear classifiers for true splicing junction detection, and achieved superior results.

In this paper, we introduced a new statistical metric, namely Minimal Match on Either Side of Exon junction (MMES), as a means to measure the “quality” of junction reads by integrating all the features listed above. Then, we presented a simple yet effective empirical statistical model using this metric to detect splicing junctions with real RNA-seq data. When validated by two highly reliable mouse transcript databases, this MMES based empirical method is shown to be remarkably more accurate than read-counting method, and also better than the logistic regression method used in Pan *et al* [3].

## Results

### 1) Overview of MMES

**Figure 1:**
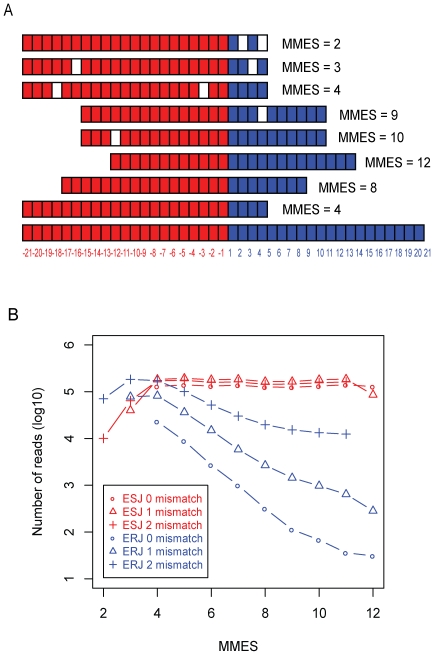
MMES metric and its skewed distribution over ESJ and ERJ. (A) Calculation of Minimal Match on Either Side (MMES) of junction. Each square represents a nucleotide. The figure shows that eight 25-mer reads are aligned to a 42-mer exon junction (the bottom). The exon junction is composed of two equal parts: the left part (filled in red) is from the last 21bp of the upstream exon, and the right part (filled in blue) is from the first 21 bp of the downstream exon. Within aligned reads, the matched nucleotides are colored in either red (left) or blue (right), and the mismatch nucleotides are blank. MMES score is placed on the right side of each read. (B) MMES score (see main text) distribution on Exon Splicing Junction (ESJ, red lines) and Exon Random Junction (ERJ, blue lines). For both ESJ and ERJ, mapped reads are divided into 3 categories: 0 mismatch (circle), 1 mismatch (triangle) and 2 mismatches (cross).

The MMES score for each junction read is calculated as:
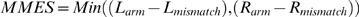
where *L_arm_* and *R_arm_* correspond to the left and right portions of the read split by joint point of exon junction, respectively. While *L_mismatch_* and *R_mismatc_*_h_ are number of mismatches occurred on *L_arm_* and *R_arm_*, respectively (Figure 1A).

For each junction read, the MMES score captured the criteria 2–4 listed above in an integrative manner. First, a junction read with fewer mismatches will have a higher MMES score. Second, a junction read with its center closer to the junction itself will have a higher MMES score. Finally, MMES can give a rough estimate of the positions of mismatches: When a read was divided into “long arm” and “short arm” by the middle point of exon junction, in most cases, mismatches on “long arm” have no effect on MMES score, while mismatches on “short arm” will reduce the MMES score. In summary, MMES is an integrated metric for measuring mapping quality, indicating the combinatorial effect of the position of the read relative to the junction and the position of the mismatch(es) in the alignment.

### 2) Non-Uniform Distribution of MMES Scores

We mapped a real RNA-seq dataset (>200M 25 bp reads) from mouse brain, liver and muscle [13] to both Exon Spliced Junction (ESJ, see Methods) and, as a negative control, Exon Random Junction (ERJ, see Methods) databases using SOAP (v1.11) with up to 2 mismatches allowed [14]. Although easy to calculate and informative, MMES is somewhat less powerful to discern different number of mismatches (i.e. 1-mismatch vs 2-mismatch). For example, additional mismatch on “long arm” has no effect on MMES score. Therefore, we divided all junction reads into 3 categories (0-mismatch, 1-mismatch and 2-mismatch), and then calculated MMES score distribution for each category, respectively. (Figure 1B). Grouping junction reads according to mismatches is necessary, because number of mismatches have great impact on mapping specificity, especially for shorter reads. This classification was also performed in previous ChIP-seq study [15].

In general, there were much more reads aligned to ESJ than to ERJ. Reads aligned to ESJ were almost uniformly distributed regardless of MMES score. In contrast, reads mapped to negative control ERJ presented a skewed distribution, i.e. the number of mapped reads drops dramatically with the increase of MMES score, which indicated that there were only a few reads having their centers close to exon junctions. Furthermore, for exact match (0-mismatch), several orders of magnitude more reads map to ESJ than to ERJ regardless of the MMES scores, which implied that exact match reads were the most reliable to detect spicing junction, no matter which part of the junction the read was aligned to. However, if there were mismatches especially 2-mismatches, nearly the same number of reads (with small MMES scores) will map to ESJ as well as ERJ (Table S1). This strongly indicated that the reliability of 2-mismatch read alignment largely depended on the corresponding MMES score, i.e. those with large MMES score were more likely to be real ones while those with small MMES score were non-specific mapping artifacts. The latter cannot be applied to identify splicing junctions without further calibration.

We also performed the same analysis on another RNA-seq dataset from human embryonic kidney and B cell line [12], and obtained very similar results (Figure S1, Table S1). We further demonstrated that this non-uniform distribution of MMES is not due to the uniqueness of read mapping, as similar distributions were observed in both uniquely and non-uniquely matched junction reads, for both human and mouse datasets (Figure S2).

### 3) Error Rate for Exon Junction Detection

The error rate of a read mapping to an exon junction (*P*_read_) can be empirically estimated from RNA-seq reads themselves, based on the MMES score distributions on ESJ (as observation) versus ERJ (as negative control).
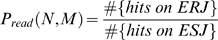
where N is MMES score and *M* is number of mismatches (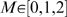 if maximum 2 mismatches allowed). Finally, the ‘pseudo’ p-value (thereafter p-value) of the exon junction (*P*_junction_) was calculated as the product of mapping error rates from all junction reads. A p-value threshold was subsequently selected (based on FDR) to call all the true exon junctions.
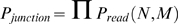



In conclusion, this empirical p-value is a measurement of reliability of junction. It was calculated based on collective effect of mapped reads, and different read had different weight according to its “mapping quality”. The smaller p-value indicates the better reliability.

### 4) Comparison with Read-Counting Method

**Figure 2:**
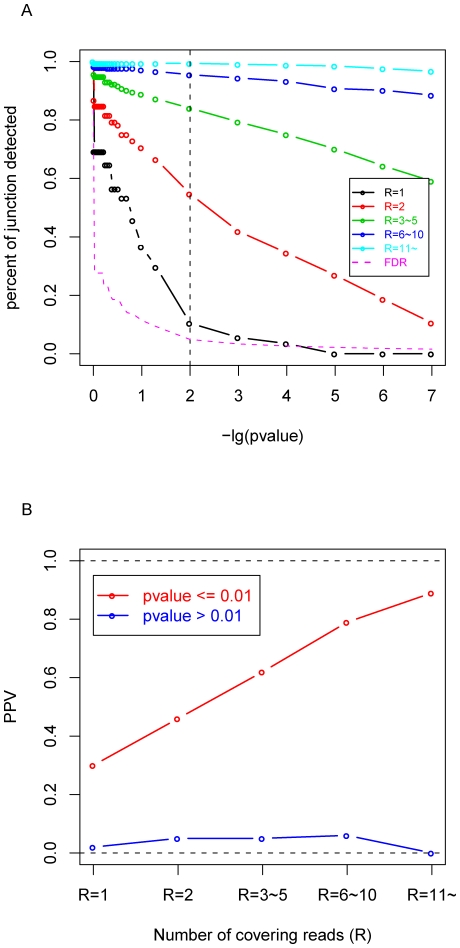
Performance of MMES based empirical method. (A) Relationship between “percent of splicing junctions detected” and p-value cut-off threshold. Splicing junctions are grouped by the number of covering reads R, the pink line indicates the incurred FDR when the corresponding cutting-off p-value is selected, and the vertical dashed line indicates the p-value = 0.01 cutoff (with incurred FDR = 4.8%). (B) In case of p-value threshold = 0.01, all junctions are divided into two classes: those junctions with p-value≤0.01 are predicted to be real, while those junctions with p-value>0.01 are predicted to be false. Each class is further divided into 5 sub-classes according to number of covering reads. For each sub-class, percent of junctions verified (PPV) is calculated by cross validating predicted junctions with combined alternative splicing database.

One drawback of read-counting method was that it failed to consider the detailed information of alignment such as mapping position and number of mismatches, and therefore, assigned all mapped reads the same weight. Another major problem was that it was difficult to set a reasonable threshold *R*, as the minimum number of covering reads with which a splicing junction would be accepted. Because as shown in Figure 2A, almost every *R* threshold in read-counting method presented a wide dynamic range of p-value inferred from our statistical method. For example, 45% of 2-read-covering junctions (*R* = 2) were rejected by our statistical method (p-value>0.01), while 55% was accepted (p-value≤0.01). From Figure 2B, we can see that on average 46% (this is the under-estimated percentage, see Discussion) of junctions with p-value≤0.01 can be verified by transcripts database, while only less than 5% of junction with p-value>0.01 can be verified.

Here we used 0.01 as a p-value cutoff, since the corresponding false discovery rate (FDR) is less than 5% (Figure 2A). The FDR was estimated by applying the same criteria to our negative control database (ERJ).

**Figure 3:**
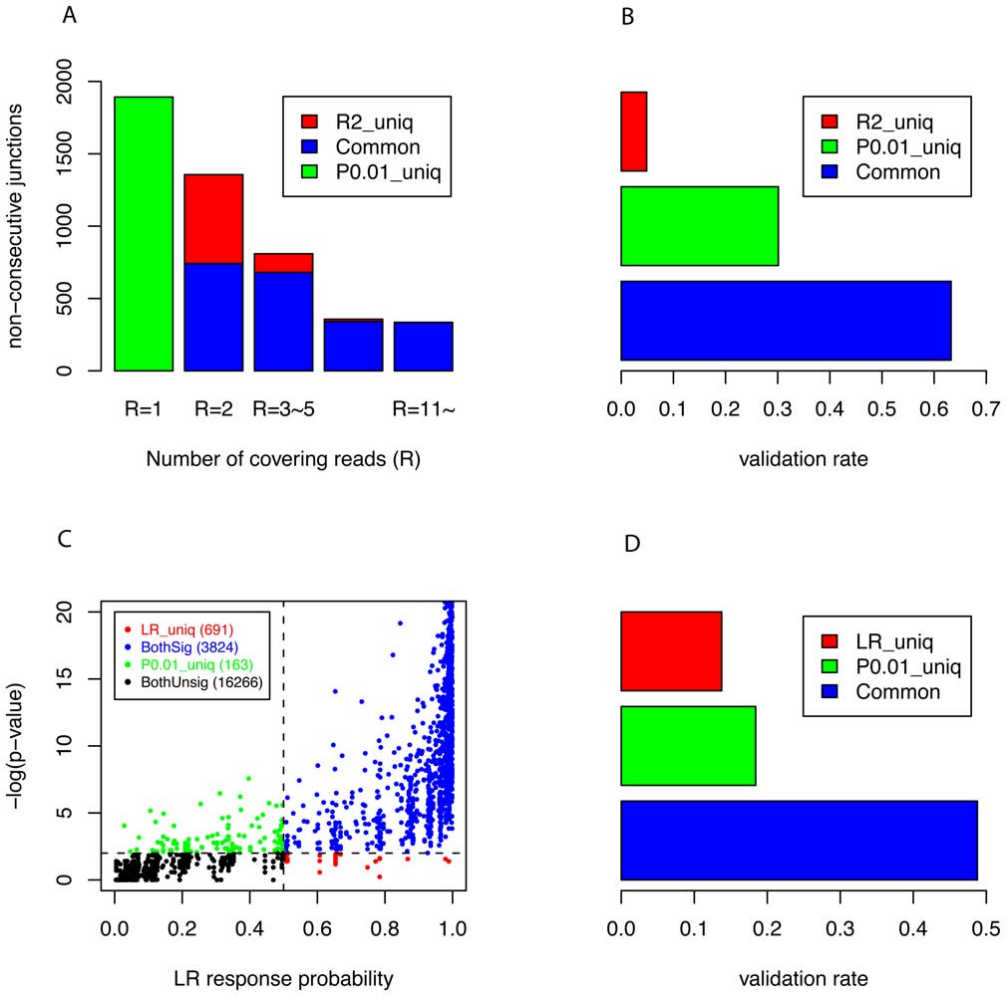
Comparison of MMES based empirical approach with read-counting method and logistic regression model. (A) All splicing junctions predicted by either method are divided into 3 non-overlapping categories: “P0.01_uniq” refers to those junctions with only 1 covering read but with p-value≤0.01 (green); “R2_uniq” refers to junctions with at least 2 covering reads but with p-value>0.01 (red). “Common” refers to those junctions with at least 2 covering reads and with p-value≤0.01 (blue). (B) Validation rate (PPV) for “P0.01_uniq”, “R2_uniq”, and “Common”, respectively. (C) “P0.01_uniq” refers to those junctions detected by MMES based empirical method only (green), “LR_uniq” refers to those junctions identified by logistic regression only (red), “BothSig” refers to junctions identified by both models (blue) and “BothUnsig” refers to junctions rejected by both method. (D) Validation rate (PPV) for “P0.01_uniq”, “LR_uniq”, and “Common”, respectively.

To further confirm that our empirical method performed better than read-counting method, we divided all splicing junctions predicted either by read-counting or our MMES based method into 3 non-overlapping categories: MMES model uniquely predicted (“P_0.01__uniq”, p-value≤0.01, *R* = 1), read-counting method uniquely (“R_2__uniq”, p-value>0.01, *R*≥2), and predicted by both methods (“Common”, p-value≤0.01, *R*≥2) (Figure 3A). For each group, we then checked the validation rate (i.e. positive predictive value; *PPV*, see discussion), using highly reliable Combined Alternative Splicing Database (CASD, see Methods) as positive control. The results indicated that 63.26% of the “Common” and 30.13% of “P_0.01__uniq” splicing junctions could be rediscovered in CASD, while only 4.86% of “R_2__uniq” splicing junctions were observed in CASD (Figure 3B). The *PPV* difference between “R_2__uniq” and “P_0.01__uniq” was statistically significant with Fisher's Exact Test p-value = 3.3×10^−54^. To our surprise, the low *PPV* (4.86%) of those R_2_ unique splicing junctions were almost constant regardless of the number of junction reads (Figure 2B), although *PPV* of the common splicing predictions rose dramatically with the increase of the number of junction reads.

The read-counting method (with *R* = 2 cutoff) had some intrinsic problems especially in detecting splicing junctions in low-abundance transcripts, where, in most cases, only one read was mapped to a splicing junction. For example, in mouse brain tissue, 20,945 non-consecutive exon junctions (skipped junctions) were supported by RNA-seq reads, of which 86.34% (18,088/20,945) were covered only by one read (Figure S3). Not surprisingly, many of them are real exon junctions rather than false positives, because as shown in Figure 3B, 30.13% of them can be found in CASD. And we believe 30.13% was an under-estimated number as low abundance transcripts were poorly represented in CASD (see discussion).

Taking all together, we demonstrated that our MMES-based empirical statistical approach is more accurate than previous used read-counting method. In terms of method-specific predictions (i.e. junctions predicted only by one method rather than the other), our method is roughly 6× more accurate than read-counting method.

### 5) Comparison with Logistic Regression Model

Logistic regression (logit model) allows one to predict a binary outcome from a set of predictor variables, which could be continues or discrete or mix of any of these. We built a logistic regression model using the same features in Pan *et al.* (see Methods). Basically, these features incorporate all the quantity and quality information of mapped reads, and all have strong p-values for the associated regression coefficients. When performing a log-likelihood test (comparing this logit model with empty model which only had an intercept), we received a p-value = 0 (χ^2^ = 106751, df = 6), indicating this logit model as a whole fits significantly better than empty model.

We then compared our MMES based empirical method with logistic regression model the same way as before: all predicted junction by MMES based method (designated as P0.01) and logistic regression model (designated as LR) were divided into 3 non-overlapping categories: common, P0.01_uniq and LR_uniq (Figure 3C). For commonly predicted junctions, 48.78% can be verified by CASD, while for P0.01_uniq and LR_uniq junctions, 18.40% and 13.75% can be verified, respectively (Figure 3D). In terms of uniquely predicted junctions, MMES model is 1.3 times more accurate than LR model (p-value = 1.7×10^−3^). Furthermore, by using 10-fold cross-validation, we obtained highly significant and comparable AUC scores from both logistic regression model (AUC = 0.981) and our empirical method (AUC = 0.982). Finally, we added the MMES feature to this logit model to see whether MMES score is also superior when used in Pan *et al*'s approach. The results indicated that the model was indeed improved, measured by both AUC and validation rate, but still not as good as our empirical approach (Figure S4).

### 6) Experimental Validation of False Positive and False Negative

We randomly selected 20 false positive (FP) and several false negative (FN) splicing junctions for experimental validation by RT-PCR followed by Sanger sequencing (Table S2). False positive splicing junctions refer to those covered by at least 2 reads but with p-value>0.01 in our statistical method. False negative splicing junctions refer to those covered by 1 read but with p-value≤0.01. We chose splicing junctions with relatively higher RNA-seq read coverage for RT-PCR analysis. Of the 20 FPs tested, all were confirmed, yielding a validation rate of 100%. An example was shown in Figure S5. In contrast, false negative is difficult to verify with RT-PCR/Sanger sequencing because those transcripts usually have extremely low expression level. As an alternative, we analyzed the EST data in public domain and found that at least 30% of the false negatives were confirmed (see discussion). Two examples were shown in Figure S6.

## Discussion

### 1) Sensitivity, Specificity and Positive Predictive Value (*PPV*)

Sensitivity, specificity and positive predictive value (*PPV*) are all widely used statistical measures of the performance of a prediction system. By definition, sensitivity refers to the proportion of actual positives which are correctly predicted as positive; specificity is the proportion of actual negatives which are correctly predicted as negative; while PPV is the proportion of predicted positives which are actual positive.
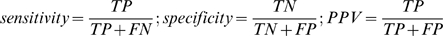
Where true positive (T*P*) refers to real exon junctions (i.e. exist in vivo) which are correctly identified as real; false positive (*FP*) refers to false exon junctions (i.e. not exist in vivo) which are incorrectly identified as real; true negative (TN) refers to false exon junctions which are correctly identified as false; and false negative (*F*N) refers to real exon junctions which are incorrectly identified as false.

Here, sensitivity and specificity cannot be applied to evaluate different methods for exon junction prediction, because we don't know the number of actual positive (T*P*+N*F*), i.e. the denominator of sensitivity, or actual negative (TN+*FP*), i.e. the denominator of specificity, in a specific tissue under a specific condition. Furthermore, some low-abundance exon junctions may not be detected just because the sequencing coverage is not deep enough, not because of the prediction method used. On the other hand, *PPV* can be determined as a statistical measure to compare our method with other method since the number of true positives (T*P*), i.e. the numerator of PPV, is confirmed by CASD, while the number of predicted positives (T*P*+*FP*), i.e. the denominator of PPV, is the result from each prediction method.

As shown in Figure 3B, 30% of the predicated splicing junctions covered by 1 read (low abundance transcripts) were confirmed by CASD. However, this does not imply that our model is less powerful in detecting low abundance transcripts. First, the *PPV* for low- abundance transcripts could be significantly under-estimated since CASD is based on traditional assays such as EST sequencing, which is much less sensitive than RNA-seq in detecting low abundance transcripts. Based on current data, it is difficult to evaluate the extent to which *PPV* is under estimated. Second, and more importantly, unlike sensitivity and specificity, which will not be affected by the ratio of positives to negatives in the sample, *PPV* suffers greatly from this ratio, and because of this, the *PPV* value *per se* cannot reflect the detecting power of a method [16].

### 2) Influence Factors for the Mapping Specificity of Junction Reads

As shown in Figure S2 and Table S1, non-specific mapping to exon junctions is a big problem for RNA-seq. In mouse dataset, 4,102,511 reads were mapped to ESJ, while 929,756 reads were mapped to negative control ERJ. Therefore as high as 23% (929,756/4,102,511) of those junction mappings are false positives. In this paper, we showed that Minimal Match on Either Side of exon junction (MMES) and the number of mismatches have very strong impacts on mapping specificity. To further determine the effect of other factors, such as read length, we performed the following simulation: assuming a uniform i.i.d. random model for DNA sequences, the number of random hits per junction per million reads (*X*) can be determined by a Random Model reported in Sultan *et al.* [12]. The results suggested that read length (*RL*) also has a big impact on mapping specificity (Table S3). For example, if two mismatches are allowed, *X* is 6.18×10^−9^ when *RL* = 32, which is about four orders of magnitude lower than that (4.44×10^−5^) when *RL* = 25. In general, other than increasing MMES and reducing the allowable mismatches, one can also increase the length of RNA-seq read to improve the mapping specificity.

### 3) Negative Control Databases

The mapping specificity of junction reads largely depends on the negative control database. Pure random sequence (PRS) shuffles the transcriptome in nucleotide level and therefore is definitely not a good control as it failed to consider inherent codon, dinucleotide and other compositional features. In contrast, ERJ and rESJ shuffle the transcriptome in exon level (i.e. the basic unit of shuffling is exon rather than nucleotide), and therefore these two databases could inherently retain most of the compositional features. As shown in Figure S7, both ERJ and rESJ reserve the compositional features of ESJ in terms of single nucleotide distribution. For example, the 5-mer motif near junction site in ESJ, i.e. 5′-[A|C]_−3_A_−2_G_−1_G_1_T_2_ -3′, can be found in both ERJ and rESJ. ERJ and rESJ can also preserve the compositional features in dinucleotide level (Table S4).

As expected, ERJ and rESJ almost give the same results when serving as negative control. In terms of mapped reads, the *Pearson's correlation coefficient* between ERJ and rESJ is 0.987 (p = 3.60×10^−14^), 0.986 (p = 5.75×10^−14^) and 0.992 (p = 8.88×10^−14^) for exact mapping, 1 mismatch mapping and 2 mismatch mapping, respectively (Figure S8).

In conclusion, both ERJ and rESJ are good negative controls because they keep compositional features inherently, however, ERJ is slightly more conservative in discriminating real splicing junctions when there are mismatches (Figure S8).

### 4) Conclusions

MMES is an integrated metric for measuring mapping quality, indicating the combinatorial effect of the position of the read relative to the junction and the position of the mismatch(es) in the alignment. Our MMES-based empirical statistical model is an annotation-based method, which relies on junction databases according to certain known gene model, and as shown in results, outperformed previous methods in terms of method-specific junction predictions. We believe that our MMES model provided a timely contribution to the splicing detection using RNA-seq.

## Materials and Methods

### 1) RNA-Seq Datasets

Two RNA-seq datasets from mouse and human were used in this study. Mouse dataset consists of 215 million, 25-bp reads from brain, liver and muscle. We downloaded the data from http://woldlab.caltech.edu/~alim/RNAseq/ [13]. Human dataset contains 13 million, 27-bp RNA-seqs from embryonic kidney and B cell line. We downloaded these data from NCBI Gene Expression Ominibus (www.ncbi.nlm.nih.gov/geo) with Accession Number GSE11892 [12].

### 2) Exon Junction Sequences

We prepared several databases in this study—ESJ (Exon Spiced Junction), ERJ (Exon Random Junction), rESJ (reversed Exon spliced Junction). We used ESJ database to detect all the potential exon skipping events. The ERJ and rESJ databases were used as negative control with the merits of maintaining inherent codon, dinucleotide and other possible compositional biases when discriminating between true and false junctions. As negative controls, ERJ and rESJ almost came up with the same results, so we only used ERJ in this study (see discussion).

ESJ database was prepared by pairwise connection of exon sequences from every locus annotated by UCSC knownGene model (mm9, July, 2007). The last 21 bp of the upstream exon was connected to the first 21 bp of the corresponding downstream exon. We tried all possible combinations, e.g. exon *i* was connected to exon *i*+1, exon *i*+2, etc. The 21-bp was chose to ensure at least 4 nucleotides overlapping between one of the two connected exons and a RNA-seq read. In our simulation, using a criterion with less than 4 nucleotides overlapping will introduce extraordinary non-specific RNA-seq read mapping to exon junctions (data not shown). After discarding those junctions shorter than reads and removing redundancy, we built the ESJ database containing 1,976,416 possible junctions.

ERJ database was constructed exactly the same way as we described for ESJ, except that two exons joined together were randomly picked from 2 different loci. For our own convenience, the sizes (i.e. total number of exon junctions) of 2 databases were set to be the same. rESJ database was also built exactly the same way as we built ESJ, except that we swapped the position of upstream exon and downstream exon.

For the human dataset, we built ESJ and ERJ databases with exactly the same method as we applied to mouse, based on UCSC knownGene model (hg18, Mar. 2006). We had in total 2,782,935 exon junctions for ESJ, ERJ. The read length is 27 bp in this dataset, so we connected the last 23 bp of upstream exon to the first 23 bp of the corresponding downstream exon, to make sure there is at least 4 bp overlapping between an exon and a RNA-seq read.

### 3) Known Splicing Junction Databases

We used two high quality transcript databases to evaluate the predicted junctions. These include the EBI Alternative Splicing and Transcripts Diversity database (ASTD, v1.1) (http://www.ebi.ac.uk/astd/main.html) and Mouse Gene Index database (v4.0) developed by National Institute of Aging (http://lgsun.grc.nia.nih.gov/geneindex4/index.html) [17], [18]. All mouse transcripts sequences were downloaded and combined as a Combined Alternative Splicing Database (CASD). All predicted exon junctions were mapped to CASD with up to two mismatches allowed. Percentage of junctions verified was measured by Positive Predictive Value (*PPV*, see discussion), which is defined as: 
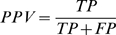
.

### 4) Logistic Regression Model

We used exact the same features in Pan *et al* [3] in their pioneering study of human transcriptome, except insertions/deletions, because none of our short 25mer reads was aligned with gaps when mapped to junctionome. Another logit model was build by adding maximum MMES score to the above features. The classifiers were trained on rESJ database (as negative) and consecutive junctions (as positive) to obtain parameters. An R package *glm* was use to implement this logistic regression and ROC curve was plot using ROCR package [19].

### 5) Experimental Validation of False Positive and False Negative

To experimentally validate the false positive and false negative splice junctions, we designed exon-specific primers and carried out Reverse Transcription Polymerase Chain Reaction (*RT-PCR*) on mouse skeletal muscle PolyA^+^ RNA. The reaction for synthesizing the 1^st^ strand cDNA consists of 2.0 µL of AccuScript RT buffer, 100 mM dNTP mix (25 mM each), 0.1µg mouse skeletal muscle polyA RNA, 100 ng of reverse exon-specific primer in a final volume of 16.5 µL. To denature the mRNA, the reactions were first incubated at 65°C for 5 min, followed by 25°C for 5 min. We then added 2.0 µL of 100 mM DTT, 1.0 µL AccuScript Reverse Transcriptase, and 2.0 Units of RNase Block ribonuclease inhibitor in that order. The reactions were incubated at 42°C for an hour, and terminated by heat inactivation at 75°C for 15 min.

To amplify the second strand cDNA, 2 µL of reverse transcription reaction product was transferred into a 50 µL PCR mixture consisting of 5 µL 10× *PfuUltra* PCR Buffer, 2.0 µL 25 mM magnesium chloride, 0.4 µL dNTP's 25 mM each, 100 ng of forward and reverse primers and 0.1 µL of *PfuUltra* Taq 2.5 U/µL. PCR cycling began with template denaturation and hot start Taq activation at 95°C for 1 min, followed by 40 cycles of 95°C for 30 sec, 55°C for 30 sec and 68°C for 3 min each, and then termianted by an extension step at 68°C for 10 min. The PCR products were analyzed on 1.5% agarose gels, and cloned into the pGEM-T TA cloning vector for sequencing. The obtained sequences were then mapped to mouse genome using UCSC genome browser for splice junction analyses. Mouse skeletal muscle PolyA^+^ RNA, *PfuUltra* II fusion HS DNA Polymerase and AccuScript High Fidelity 1^st^-Strand cDNA Synthesis Kit were purchased from Stratagene, and pGEM-T TA cloning vector from Promega.

## Supporting Information

Figure S1 MMES distribution for Human RNA-seq. Comparison of MMES distribution between Exon Splicing Junction (ESJ, red lines) and Exon Randomly Junction (ERJ, blue lines) for Human RNA-seq dataset. Mapped reads are divided into 3 categories: 0 mismatch (triangle), 1 mismatch (cross) and 2 mismatches (diamond).
(0.02 MB PDF)

Figure S2 Comparison of MMES for uniquely mapped reads (square) and non-uniquely mapped reads (triangle). (A) Mouse dataset, (B)Human dataset.
(0.29 MB PDF)

Figure S3 Pie chart of non-consecutive junctions. Nonconsecutive exon junctions (skipped junctions) are divided into 5 groups, according to number of covering reads (R).
(0.14 MB PDF)

Figure S4 ROC curve of logistic regression model. (A), (B) Receiver Operating Characteristic (ROC) curves for logistic regression model without and with MMES feature, respectively. Blue dots indicate 10 cross-validation runs, red solid line is average curve (C) Validation rates of commonly predicted junctions (“Common”, blue), MMES-based empirical method (“P0.01_uniq”, green), logistic regression model without MMES feature (“LR (MMES-) uniq”, red)” and logistic regression model with MMES feature (“LR (MMES+) uniq”, pink).
(1.28 MB PDF)

Figure S5 Examples of false positive. (A) A junction between exon2 and exon4 (the first exon is indexed as 0) of Tnnc2 (uc008nvz.1) is covered by 6 reads but with p-value = 1, and therefore is rejected by MMES statistic model (cutoff p-value = 0.01). (B) Screen shot form UCSC genome browser. We design exon specific primer pair (forward primer on exon2 and reverse primer on exon 4) and carry out RT-PCR on mouse muscle total RNA. 23 randomly picked clones from the PCR product are sequenced, without observing the anticipated junction (p<0.05).
(0.70 MB PDF)

Figure S6 Examples of false negative. (A) A junction between exon1 and exon3 of gene Polr2d (uc008eim.1) is covered by 1 read but with significant p-value = 8.9×10^−5^. (B) This junction is confirmed by 3 independent EST sequences. (C) A junction between exon1 and exon3 of gene Mcat (uc007xbf.1) is covered by 1 read but with significant p-value = 6×10^−6^. (D) This junction is supported by 2 independent EST sequences. Skipped exon is indicated with red arrow.
(0.63 MB PDF)

Figure S7 Single nucleotide frequency distribution of ESJ, ERJ and rESJ. Coordinates (5′ to 3′) of oligo from last 21bp of upstream exon are indexed from −21 to −1, and coordinates (5′ to 3′) of oligo from first 21bp of downstream exon are indexed from 1 to 21.
(0.75 MB PDF)

Figure S8 Compare mapped reads distribution among ESJ, ERJ and rESJ. For each database, mapped reads were divided into 3 categories: 0-mismatch (exact match), 1-mismatch and 2-mismatch. The mapping position of each mapped read was represented by its middle-point. (A) ESJ vs ERJ, (B) ESJ vs rESJ, (C) ERJ vs rESJ.
(0.82 MB PDF)

Table S1 Distribution of mapped reads over exon junctions. Each read is represented by its midpoint. (A) Mouse dataset. (B) Human dataset.
(0.02 MB PDF)

Table S2 List of selected false positive exon junctions and primers.
(0.03 MB PDF)

Table S3 Simulation of false positive hits based on random model. Read length varies from 10 to 50. Number of mismatch ranges from 0 to 5.
(0.02 MB PDF)

Table S4 Distribution of dinucleotide frequency across ESJ, rESJ and ERJ.
(0.07 MB PDF)
