# Supplementary figures and images for: A Statistical Method for the Detection of Alternative Splicing Using RNA-Seq
Source: PLoS One. 2010 Jan 8;5(1):e8529. doi: 10.1371/journal.pone.0008529 (PMC2798953; doi:10.1371/journal.pone.0008529)

# Human (read=27bp, junction=46bp)

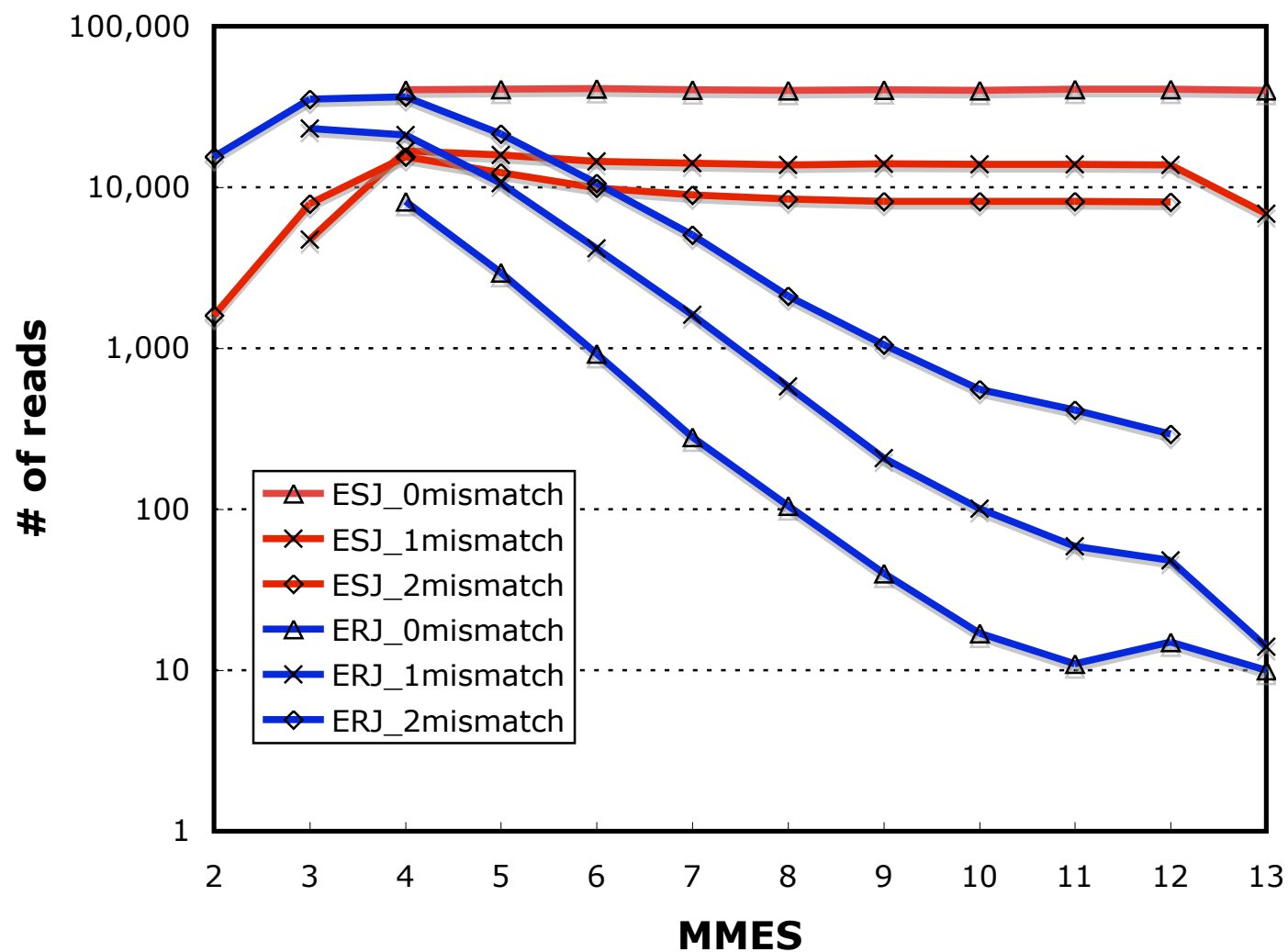

Supplement: Figure S1 — MMES distribution for Human RNA-seq. Comparison of MMES distribution between Exon Splicing Junction (ESJ, red lines) and Exon Randomly Junction (ERJ, blue lines) for Human RNA-seq dataset. Mapped reads are divided into 3 categories: 0 mismatch (triangle), 1 mismatch (cross) and 2 mismatches (diamond). (0.02 MB PDF) [file pone.0008529.s001.pdf]

(A)

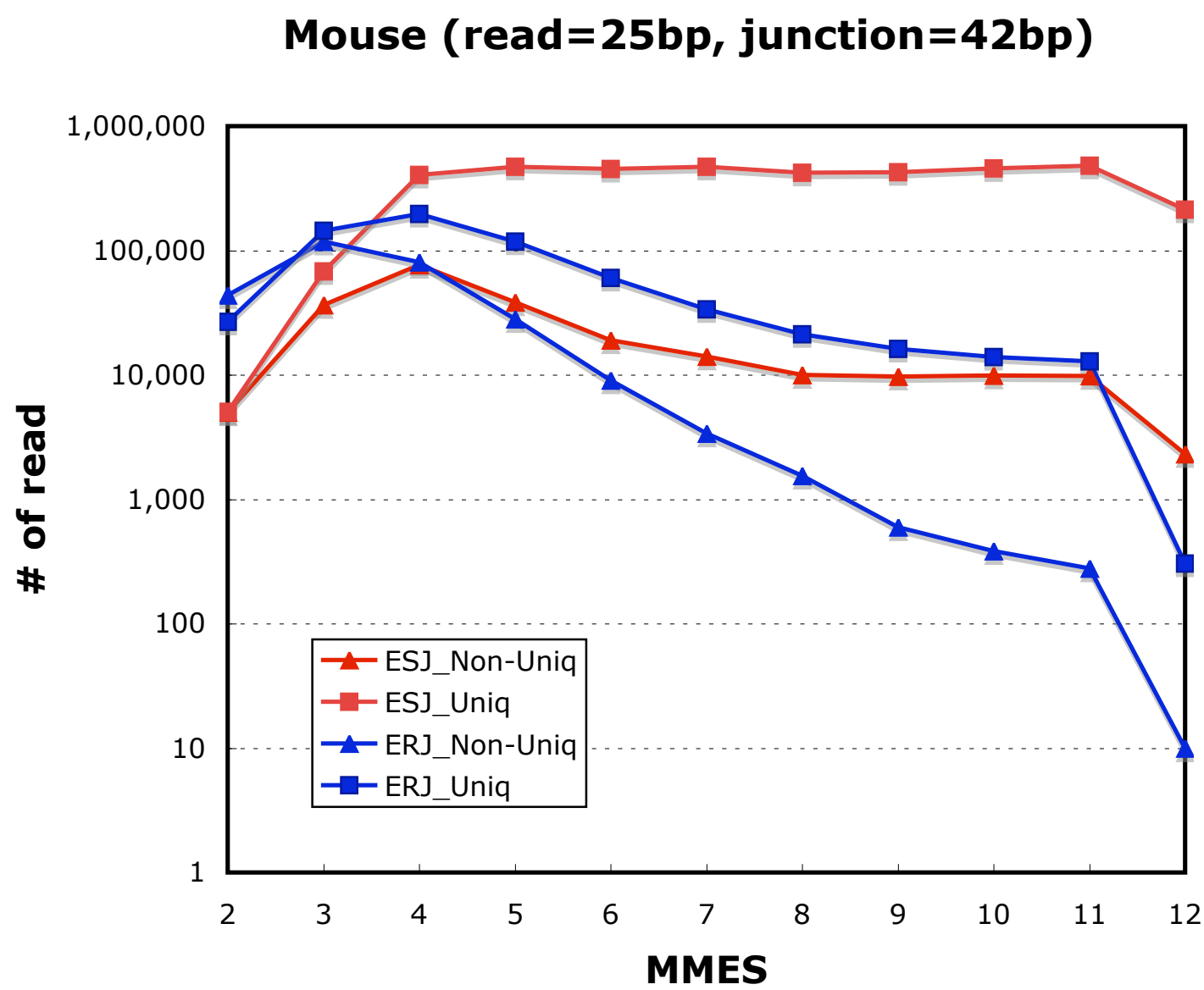

(B)

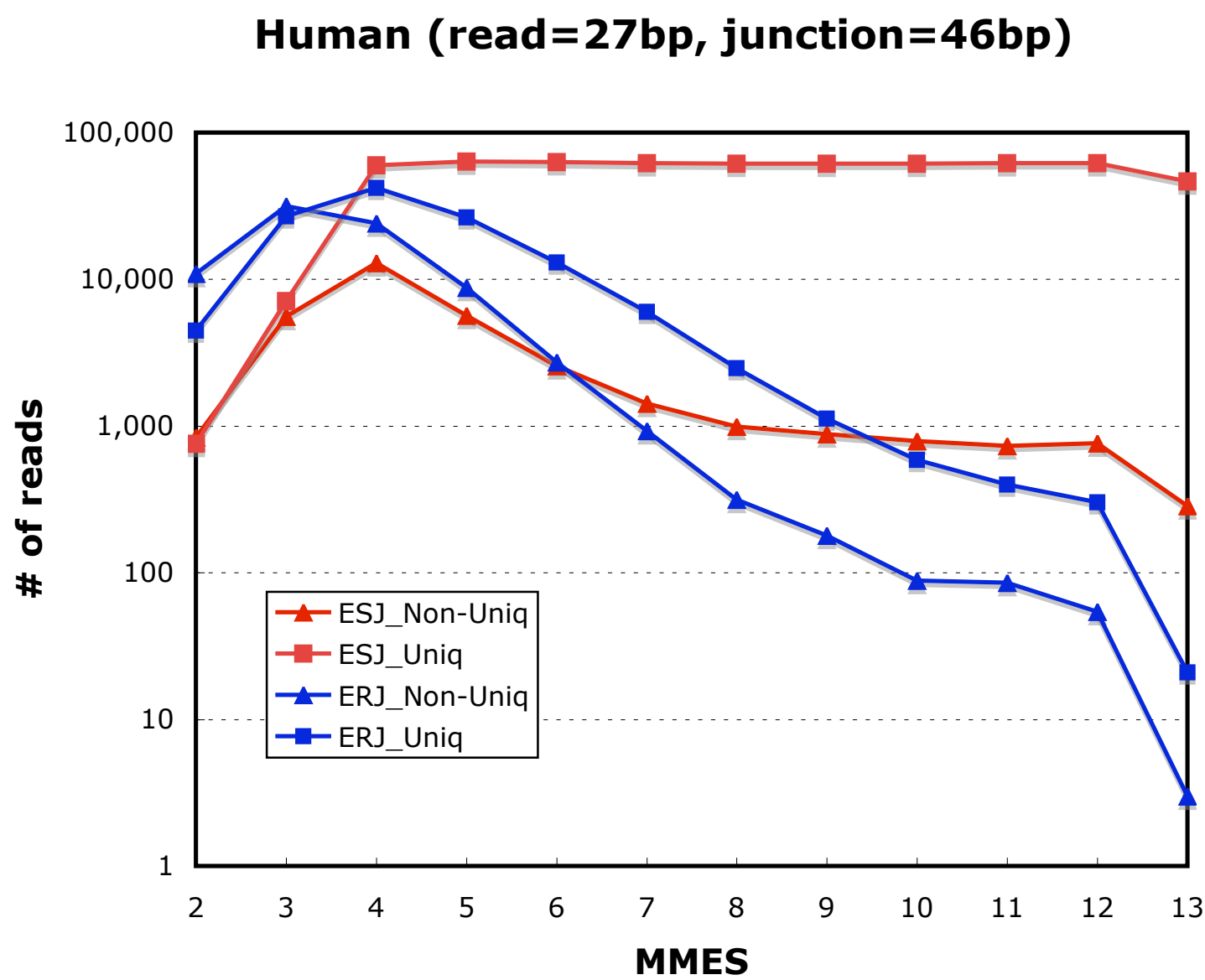

Supplement: Figure S2 — Comparison of MMES for uniquely mapped reads (square) and non-uniquely mapped reads (triangle). (A) Mouse dataset, (B)Human dataset. (0.29 MB PDF) [file pone.0008529.s002.pdf]

coverage of non-consecutive junctions

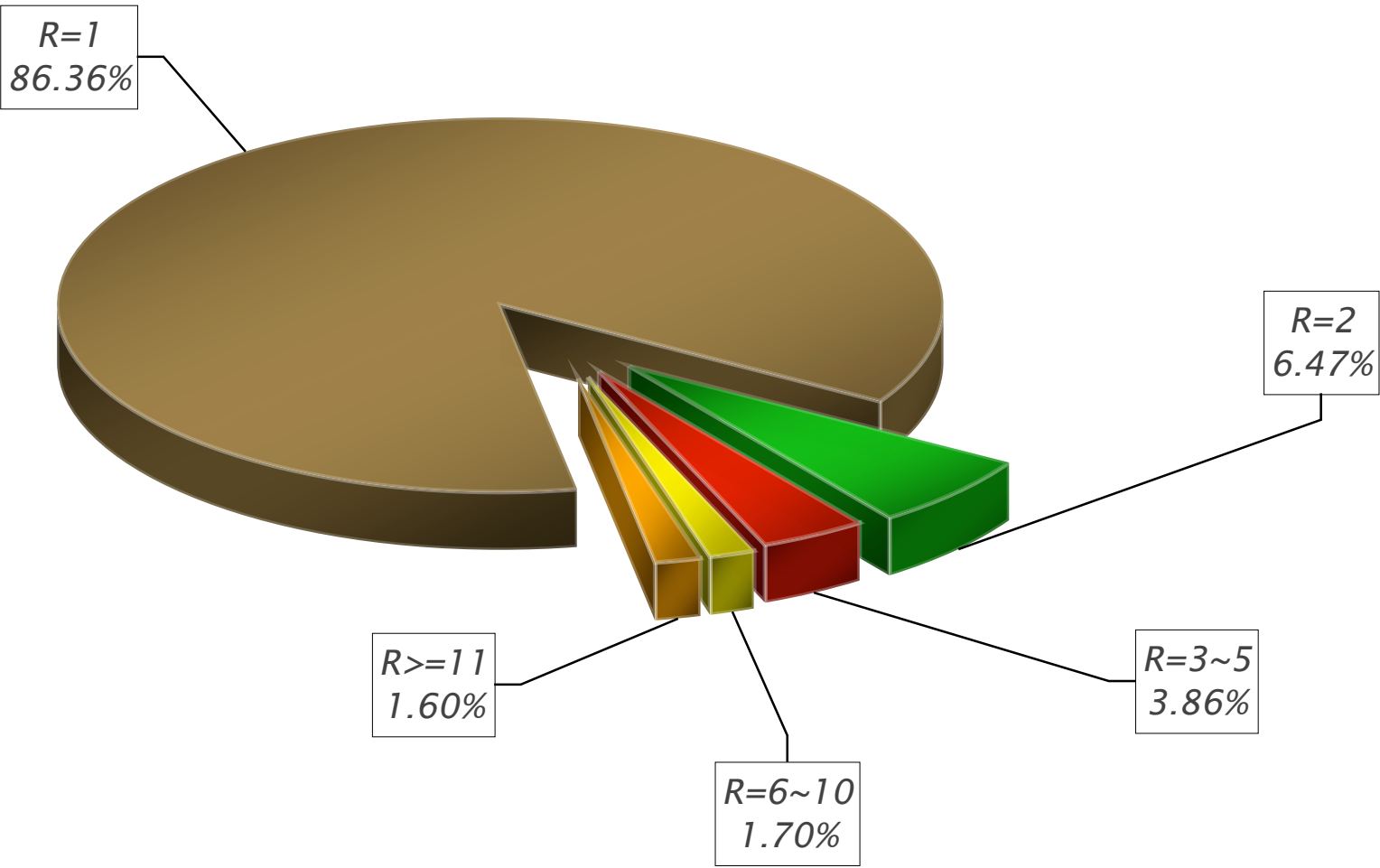

Supplement: Figure S3 — Pie chart of non-consecutive junctions. Nonconsecutive exon junctions (skipped junctions) are divided into 5 groups, according to number of covering reads (R). (0.14 MB PDF) [file pone.0008529.s003.pdf]

A **MMES-**

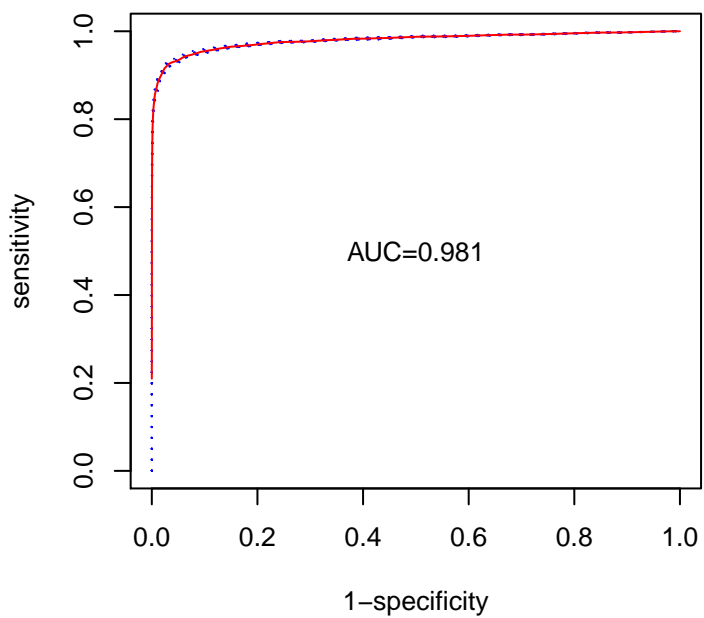

B **MMES+**

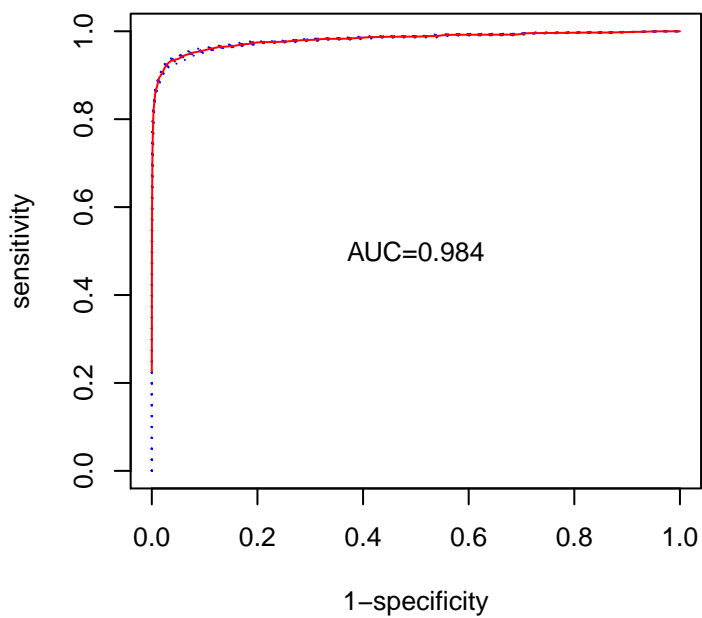

C

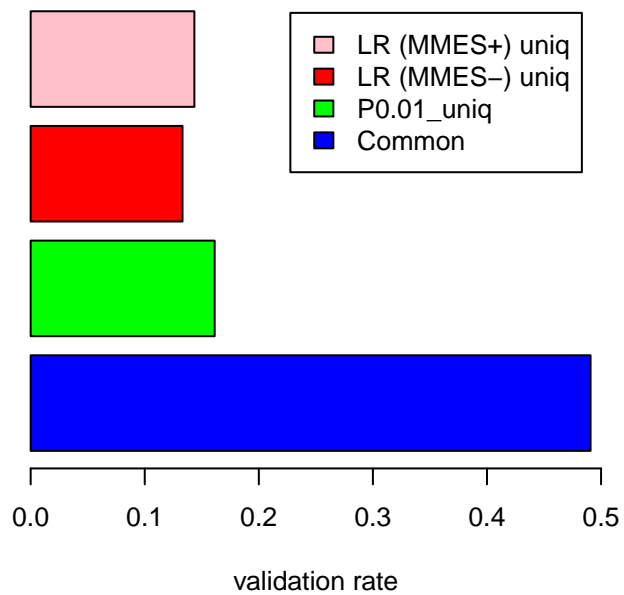

Supplement: Figure S4 — ROC curve of logistic regression model. (A), (B) Receiver Operating Characteristic (ROC) curves for logistic regression model without and with MMES feature, respectively. Blue dots indicate 10 cross-validation runs, red solid line is average curve (C) Validation rates of commonly predicted junctions (“Common”, blue), MMES-based empirical method (“P0.01_uniq”, green), logistic regression model without MMES feature (“LR (MMES-) uniq”, red)” and logistic regression model with MMES feature (“LR (MMES+) uniq”, pink). (1.28 MB PDF) [file pone.0008529.s004.pdf]

**A**

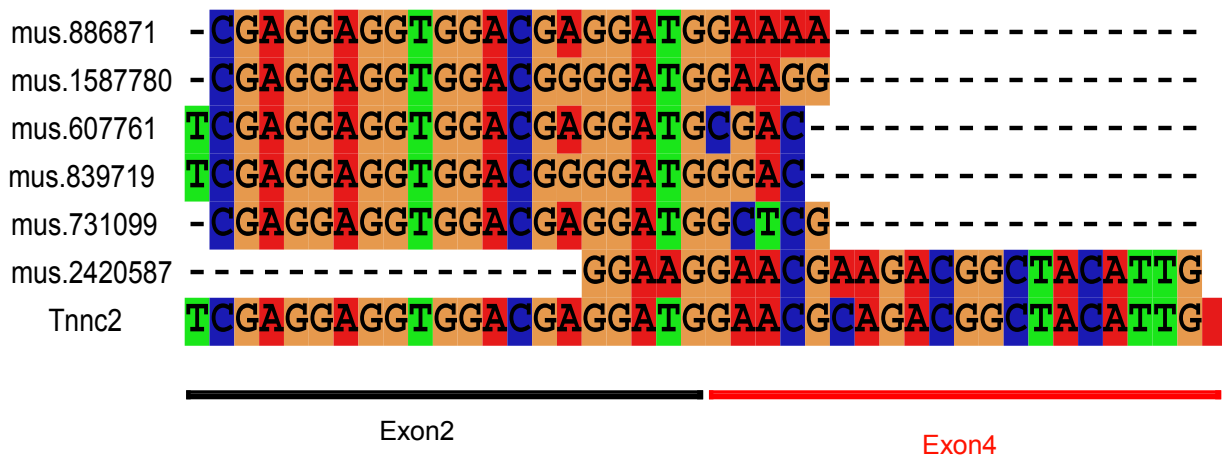

**B**

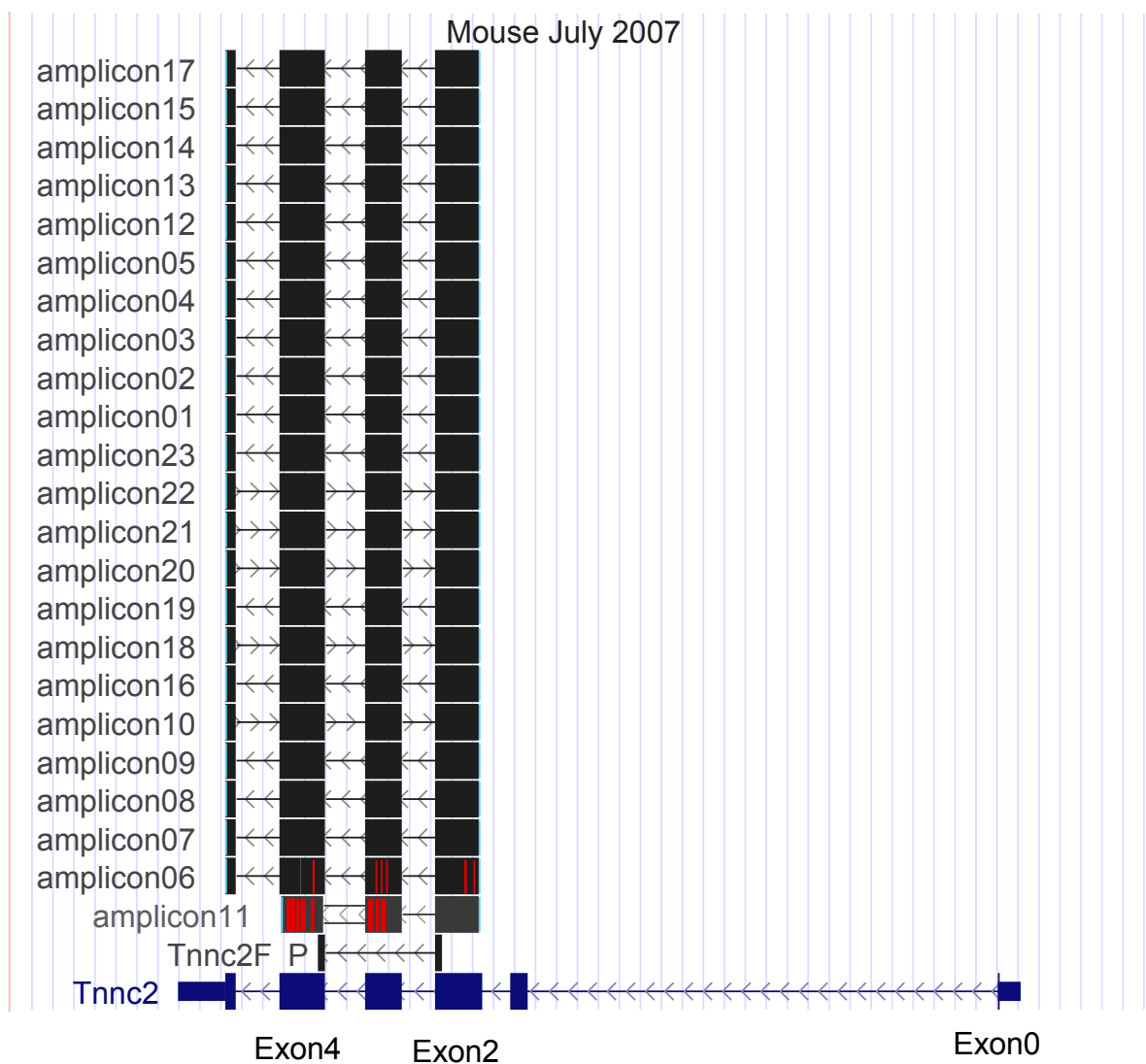

Supplement: Figure S5 — Examples of false positive. (A) A junction between exon2 and exon4 (the first exon is indexed as 0) of Tnnc2 (uc008nvz.1) is covered by 6 reads but with p-value = 1, and therefore is rejected by MMES statistic model (cutoff p-value = 0.01). (B) Screen shot form UCSC genome browser. We design exon specific primer pair (forward primer on exon2 and reverse primer on exon 4) and carry out RT-PCR on mouse muscle total RNA. 23 randomly picked clones from the PCR product are sequenced, without observing the anticipated junction (p<0.05). (0.70 MB PDF) [file pone.0008529.s005.pdf]

**A**

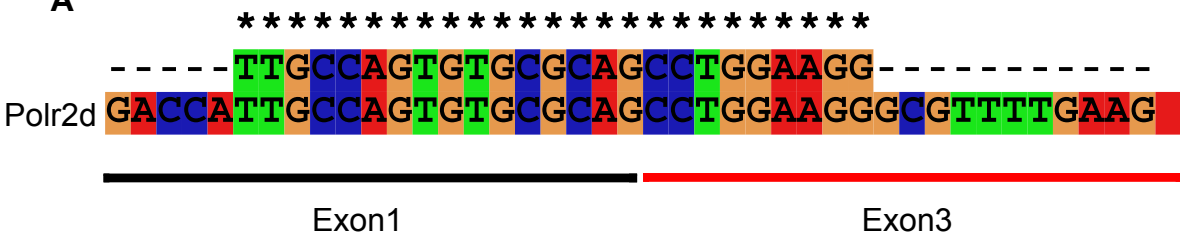

**B**

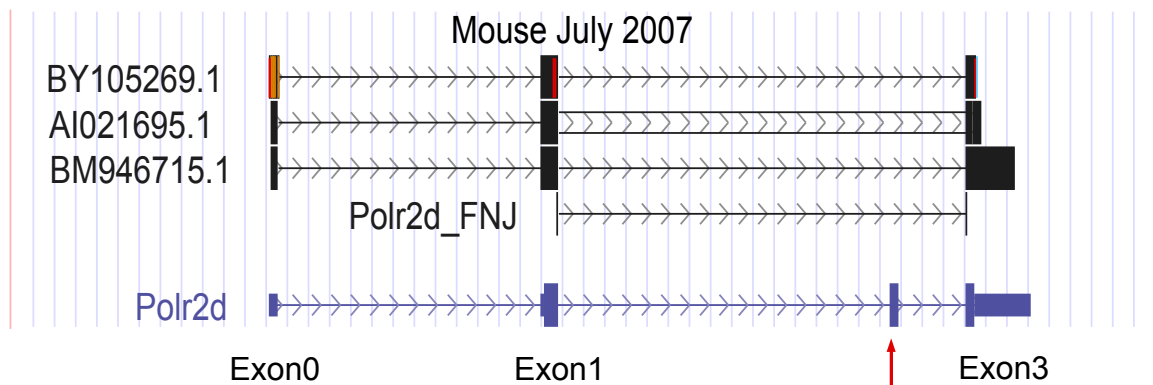

**C**

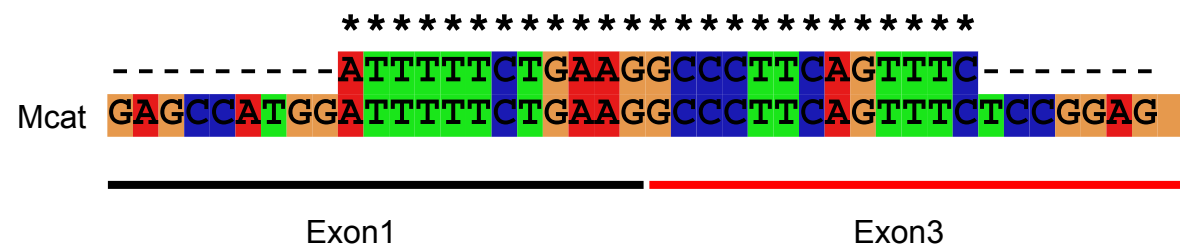

**D**

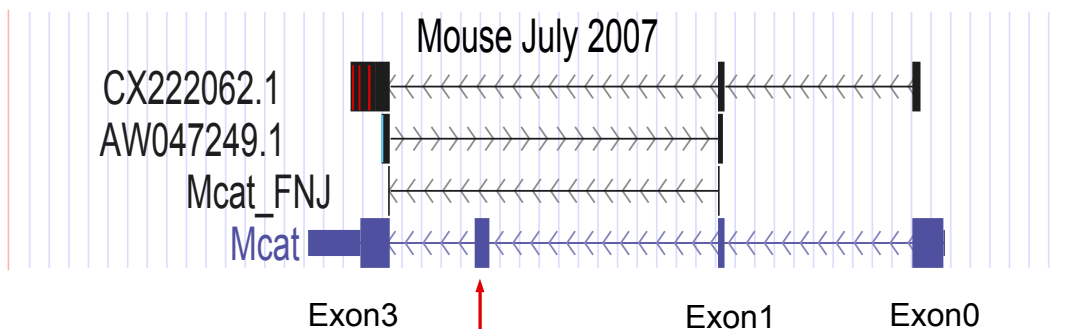

Supplement: Figure S6 — Examples of false negative. (A) A junction between exon1 and exon3 of gene Polr2d (uc008eim.1) is covered by 1 read but with significant p-value = 8.9×10^−5^. (B) This junction is confirmed by 3 independent EST sequences. (C) A junction between exon1 and exon3 of gene Mcat (uc007xbf.1) is covered by 1 read but with significant p-value = 6×10^−6^. (D) This junction is supported by 2 independent EST sequences. Skipped exon is indicated with red arrow. (0.63 MB PDF) [file pone.0008529.s006.pdf]

**A**

ESJ vs ERJ

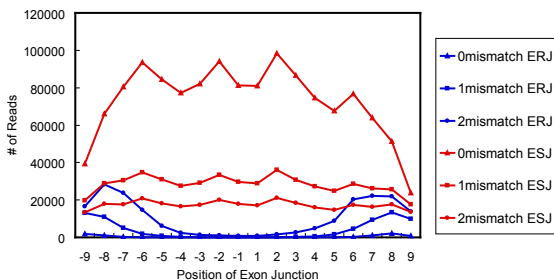**B**

ESJ vs rESJ

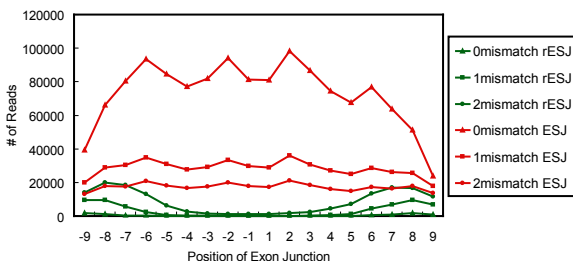**C**

ERJ vs rESJ

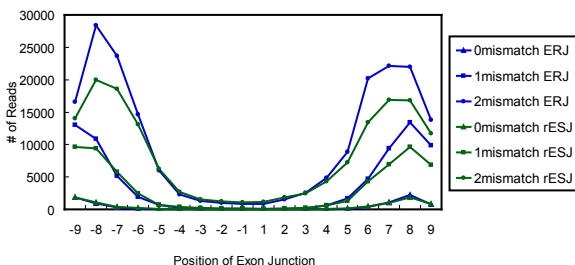

Supplement: Figure S8 — Compare mapped reads distribution among ESJ, ERJ and rESJ. For each database, mapped reads were divided into 3 categories: 0-mismatch (exact match), 1-mismatch and 2-mismatch. The mapping position of each mapped read was represented by its middle-point. (A) ESJ vs ERJ, (B) ESJ vs rESJ, (C) ERJ vs rESJ. (0.82 MB PDF) [file pone.0008529.s008.pdf]
