# Supplementary material for: A Statistical Method for the Detection of Alternative Splicing Using RNA-Seq
Source: PLoS One. 2010 Jan 8;5(1):e8529. doi: 10.1371/journal.pone.0008529 (PMC2798953; doi:10.1371/journal.pone.0008529)
Supplement: Figure S7 — Single nucleotide frequency distribution of ESJ, ERJ and rESJ. Coordinates (5′ to 3′) of oligo from last 21bp of upstream exon are indexed from −21 to −1, and coordinates (5′ to 3′) of oligo from first 21bp of downstream exon are indexed from 1 to 21. (0.75 MB PDF) [file pone.0008529.s007.pdf]

A

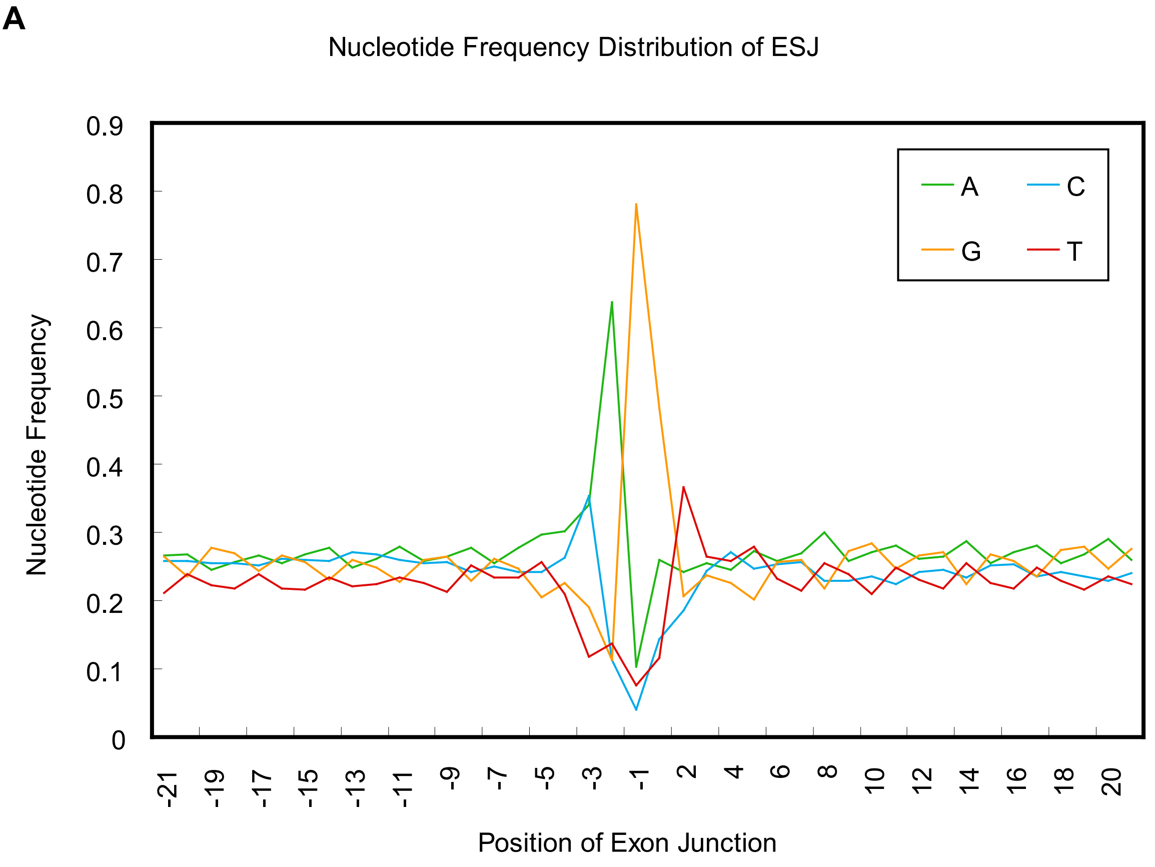
Nucleotide Frequency Distribution of ESJ
Nucleotide Frequency
Position of Exon Junction
A
C
G
T

B

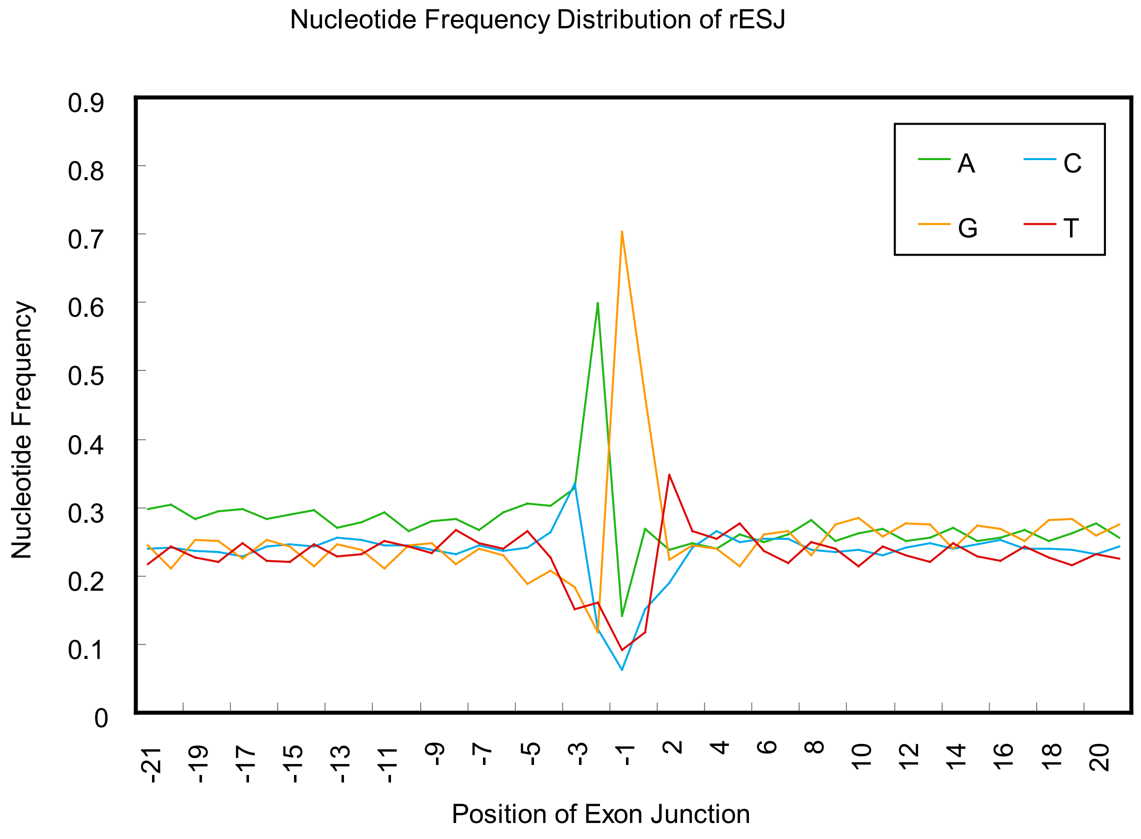
Nucleotide Frequency Distribution of rESJ
Nucleotide Frequency
Position of Exon Junction
A
C
G
T

C

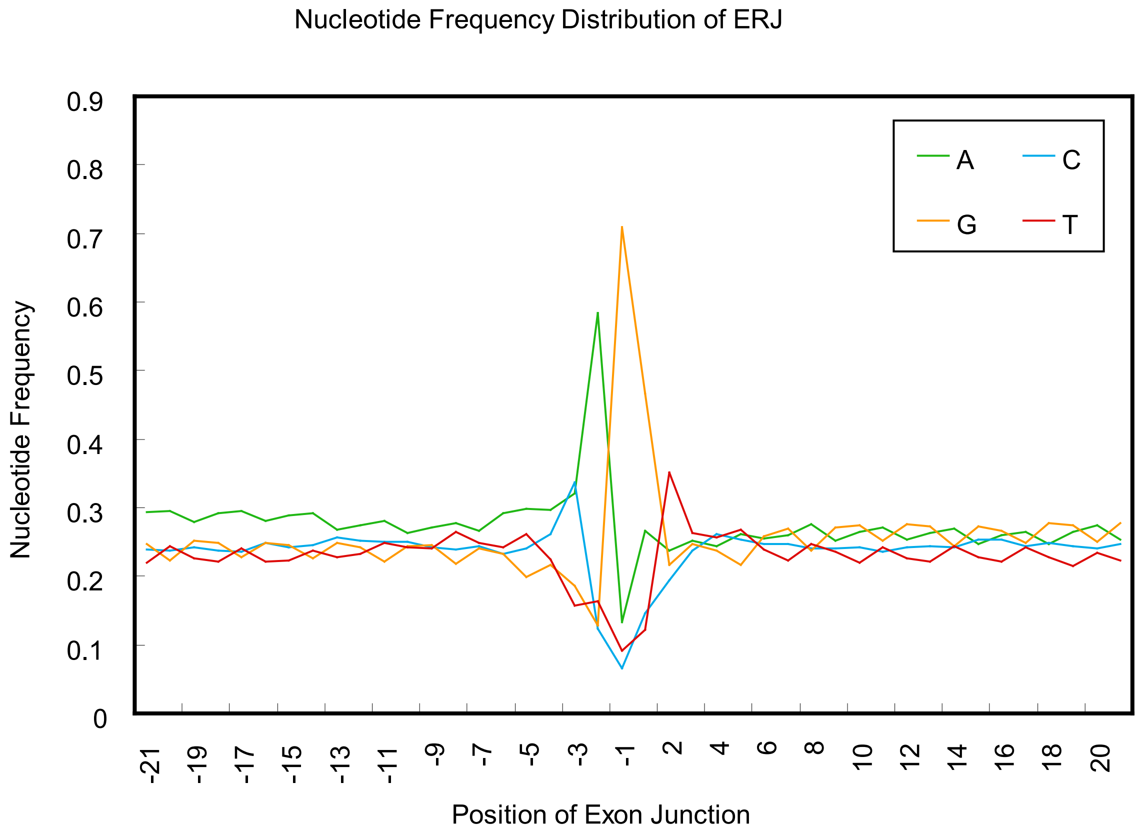
Nucleotide Frequency Distribution of ERJ
Nucleotide Frequency
Position of Exon Junction
A
C
G
T
