# Supplementary material for: A Statistical Method for the Detection of Alternative Splicing Using RNA-Seq
Source: PLoS One. 2010 Jan 8;5(1):e8529. doi: 10.1371/journal.pone.0008529 (PMC2798953; doi:10.1371/journal.pone.0008529)
Supplement: Table S1 — Distribution of mapped reads over exon junctions. Each read is represented by its midpoint. (A) Mouse dataset. (B) Human dataset. (0.02 MB PDF) [file pone.0008529.s009.pdf]

Table S1 A Mapping distribution over mouse exon junctions

Position of ESJ/ERJ	# of reads on ESJ (0mismatch)	# of reads on ESJ (1mismatch)	# of reads on ESJ (2mismatch)	# of reads on ERJ (0mismatch)	# of reads on ERJ (1mismatch)	# of reads on ERJ (2mismatch)
**-9**	59,903	77,254	69,064	5,666	42,314	93,168
**-8**	67,659	92,105	81,193	2,198	23,144	83,431
**-7**	67,129	88,703	74,866	701	9,493	50,763
**-6**	72,090	98,040	85,596	263	4,175	31,019
**-5**	66,302	87,085	74,572	93	1,722	16,725
**-4**	62,455	78,548	65,785	32	786	9,603
**-3**	65,273	83,024	67,842	20	442	7,369
**-2**	69,370	93,088	78,713	20	405	6,959
**-1**	64,617	83,670	70,853	18	310	6,195
**1**	64,043	82,562	70,112	13	295	5,896
**2**	71,532	96,224	82,503	16	505	7,792
**3**	66,656	87,551	74,045	48	857	9,709
**4**	62,292	78,125	67,000	81	1,701	14,592
**5**	59,379	73,630	63,126	224	3,323	22,848
**6**	65,744	86,024	75,520	731	8,595	42,664
**7**	64,872	84,419	75,259	2,005	19,564	65,119
**8**	72,217	98,716	88,733	6,660	42,341	93,601
**9**	69,845	94,131	83,452	17,338	63,414	102,790

Table S1 B Mapping distribution over human exon junctions

Position of ESJ/ERJ	# of reads on ESJ (0mismatch)	# of reads on ESJ (1mismatch)	# of reads on ESJ (2mismatch)	# of reads on ERJ (0mismatch)	# of reads on ERJ (1mismatch)	# of reads on ERJ (2mismatch)
**-10**	19,444	7,547	5,821	2,087	12,749	17,956
**-9**	20,195	7,595	5,954	695	7,109	17,319
**-8**	20,169	7,299	5,298	247	3,084	12,028
**-7**	20,202	6,828	4,591	75	1,179	6,723
**-6**	20,203	7,048	4,597	43	491	3,237
**-5**	20,135	6,656	3,845	16	158	1,499
**-4**	19,856	6,714	3,671	8	79	638
**-3**	20,364	6,699	3,764	6	41	377
**-2**	20,265	6,789	3,803	8	27	252
**-1**	19,846	6,594	3,798	2	18	199
**1**	20,183	6,858	3,861	8	25	178
**2**	20,562	6,847	3,953	7	29	294
**3**	20,176	6,722	4,146	5	51	542
**4**	20,199	6,905	4,131	9	124	1,102
**5**	20,051	6,911	4,202	24	345	2,474
**6**	19,905	6,509	4,400	62	924	5,155
**7**	20,278	7,050	5,029	207	2,526	9,804
**8**	20,727	7,457	6,120	680	6,217	14,857
**9**	20,486	8,260	7,591	2,251	11,219	17,446
**10**	20,892	8,672	8,708	6,074	15,377	16,990
