# Supplementary material for: A Statistical Method for the Detection of Alternative Splicing Using RNA-Seq
Source: PLoS One. 2010 Jan 8;5(1):e8529. doi: 10.1371/journal.pone.0008529 (PMC2798953; doi:10.1371/journal.pone.0008529)
Supplement: Table S2 — List of selected false positive exon junctions and primers. (0.03 MB PDF) [file pone.0008529.s010.pdf]

Label	Clone_ID	ExonJunction	Primer_F (5' ->3')	Primer_R (5' ->3')
FP	1	uc007jmj.1_26-38	TGAAGCAGAAGCTGGAGAAGGAGA	CTTGGCCAGGTTGACATTGGATTG
FP	2	uc007wov.1_1-3	AAGAAGGCGATAGGAGCCTTTGCT	TTCCAGAAGAATGGCGTCATCCGA
FP	3	uc008gbf.1_0-12	AGCCAAGAGCCTGAGCAAGATGAT	AATGGTCTCCAGGAGCTTCTCCAT
FP	4	uc008gbf.1_9-19	CAACTTCAACACGCTGCAGACCAA	TACCACTTGTTCAGCCGTGTCTGT
FP	5	uc008gio.1_9-11	ACGTAACAGTGAAGACCTGTGCCT	TTTGGCCACATCACGGATAAAGGC
FP	6	uc008nue.1_0-2	TCTCTGCTTCTCCTTCTGGTGACA	CTCTTTATTGGAAAGAAAGCTCCAGAGT
FP	7	uc008nvz.1_2-4	GCTGCCTTTGACATGTTCGATGCT	ACTGAACGCCCTCCATCATCTTCA
FP	8	uc008sqd.1_2-4	AGAAACTGGAGCAGGCTGAGAAGA	GGGCTTCCAGGGATTTCAAGTTGT
FP	9	uc009jrf.1_2-10	AGACCTGCTTGTGCGGATTCTTCT	AGAGAGGCTTCTCACTTCCGTGTT
FP	10	uc009jrf.1_4-6	AGAGAACGCTATCGAAGCGCTGAA	TGGTCTCGGATCTTGCCAATCTCA
FP	11	uc009jrf.1_4-8	AAACCGTCACTGCCTTTGTGGAAC	CACGATGGCGTTCTTCTTTGCCAT
FP	12	uc009jrf.1_4-12	AGAGAACGCTATCGAAGCGCTGAA	ACGCACATAGTTACAGCGGTCGAT
FP	13	uc009jrf.1_4-22	AGAGAACGCTATCGAAGCGCTGAA	GCTTTGGCTGAAGATGCATGGCTA
FP	14	uc009jrf.1_3-15	ATCGACCGCTGTAACTATGTGCGT	ATTTCAGAGGCCGTCTTAGCCACA
FP	15	uc009jrf.1_15-22	TGTGGCTAAGACGGCCTCTGAAAT	GCTTTGGCTGAAGATGCATGGCTA
FP	16	uc009jup.1_1-5	AGGGCTAAGGTTACCACGGTATGT	GAAGGCTGCCCACATGTTCTTGAT
FP	17	uc009jup.1_2-6	ACCAGACTCAGATCCAGGAGTTCA	TCCTGGTCCTTAGCATCACCATGT
FP	18	uc009jup.1_4-6	TCGACGCTATGATGAAGGAAGCCA	TCCTGGTCCTTAGCATCACCATGT
FP	19	uc009kna.1_2-4	AGAAAGAGGAAAGCCGCCGAGAAT	AAGTGGGCAGTTAGGACTCAGACT
FP	20	uc009nwr.1_4-6	CATCACCATCGGCAATGAGCGTTT	CCTAGAAGCATTTGCGGTGCACAA
