# Supplementary material for: A Statistical Method for the Detection of Alternative Splicing Using RNA-Seq
Source: PLoS One. 2010 Jan 8;5(1):e8529. doi: 10.1371/journal.pone.0008529 (PMC2798953; doi:10.1371/journal.pone.0008529)
Supplement: Table S3 — Simulation of false positive hits based on random model. Read length varies from 10 to 50. Number of mismatch ranges from 0 to 5. (0.02 MB PDF) [file pone.0008529.s011.pdf]

Table S3

		Random hits per million reads per junction					
**Read(bp)**	**Junction (bp)**	**Mismatch=0**	**Mismatch=1**	**Mismatch=2**	**Mismatch=3**	**Mismatch=4**	**Mismatch=5**
10	12	2.86E+00	8.87E+01	1.25E+03	1.05E+04	5.92E+04	2.34E+05
11	14	9.54E-01	3.24E+01	5.04E+02	4.75E+03	3.02E+04	1.37E+05
12	16	2.98E-01	1.10E+01	1.88E+02	1.96E+03	1.39E+04	7.13E+04
13	18	8.94E-02	3.58E+00	6.63E+01	7.57E+02	5.93E+03	3.39E+04
14	20	2.61E-02	1.12E+00	2.25E+01	2.79E+02	2.39E+03	1.51E+04
15	22	7.45E-03	3.43E-01	7.38E+00	9.89E+01	9.23E+02	6.36E+03
16	24	2.10E-03	1.03E-01	2.37E+00	3.40E+01	3.43E+02	2.57E+03
17	26	5.82E-04	3.03E-02	7.43E-01	1.14E+01	1.24E+02	9.99E+02
18	28	1.60E-04	8.80E-03	2.29E-01	3.76E+00	4.34E+01	3.77E+02
19	30	4.37E-05	2.53E-03	6.97E-02	1.21E+00	1.49E+01	1.38E+02
20	32	1.18E-05	7.21E-04	2.09E-02	3.85E-01	5.02E+00	4.96E+01
21	34	3.18E-06	2.04E-04	6.22E-03	1.21E-01	1.66E+00	1.74E+01
22	36	8.53E-07	5.71E-05	1.83E-03	3.73E-02	5.42E-01	6.00E+00
23	38	2.27E-07	1.59E-05	5.34E-04	1.14E-02	1.74E-01	2.03E+00
24	40	6.04E-08	4.41E-06	1.54E-04	3.45E-03	5.54E-02	6.79E-01
25	42	1.60E-08	1.22E-06	4.44E-05	1.04E-03	1.74E-02	2.24E-01
26	44	4.22E-09	3.33E-07	1.27E-05	3.09E-04	5.42E-03	7.29E-02
27	46	1.11E-09	9.10E-08	3.60E-06	9.13E-05	1.67E-03	2.34E-02
28	48	2.91E-10	2.48E-08	1.02E-06	2.68E-05	5.10E-04	7.47E-03
29	50	7.63E-11	6.72E-09	2.86E-07	7.82E-06	1.55E-04	2.36E-03
30	52	1.99E-11	1.82E-09	7.99E-08	2.27E-06	4.66E-05	7.37E-04
31	54	5.20E-12	4.89E-10	2.23E-08	6.54E-07	1.39E-05	2.29E-04
32	56	1.36E-12	1.31E-10	6.18E-09	1.88E-07	4.14E-06	7.05E-05
33	58	3.52E-13	3.52E-11	1.71E-09	5.36E-08	1.22E-06	2.15E-05
34	60	9.15E-14	9.42E-12	4.71E-10	1.53E-08	3.59E-07	6.54E-06
35	62	2.37E-14	2.51E-12	1.30E-10	4.32E-09	1.05E-07	1.98E-06
36	64	6.14E-15	6.69E-13	3.55E-11	1.22E-09	3.05E-08	5.93E-07
37	66	1.59E-15	1.78E-13	9.70E-12	3.43E-10	8.84E-09	1.77E-07
38	68	4.10E-16	4.72E-14	2.64E-12	9.61E-11	2.55E-09	5.26E-08
39	70	1.06E-16	1.25E-14	7.19E-13	2.68E-11	7.32E-10	1.55E-08
40	72	2.73E-17	3.30E-15	1.95E-13	7.48E-12	2.10E-10	4.57E-09
41	74	7.03E-18	8.72E-16	5.28E-14	2.08E-12	5.98E-11	1.34E-09
42	76	1.81E-18	2.30E-16	1.43E-14	5.75E-13	1.70E-11	3.91E-10
43	78	4.65E-19	6.05E-17	3.84E-15	1.59E-13	4.81E-12	1.14E-10
44	80	1.20E-19	1.59E-17	1.03E-15	4.38E-14	1.36E-12	3.29E-11
45	82	3.07E-20	4.17E-18	2.78E-16	1.20E-14	3.82E-13	9.50E-12
46	84	7.88E-21	1.09E-18	7.45E-17	3.30E-15	1.07E-13	2.73E-12
47	86	2.02E-21	2.87E-19	1.99E-17	9.04E-16	3.01E-14	7.83E-13
48	88	5.17E-22	7.50E-20	5.33E-18	2.47E-16	8.40E-15	2.24E-13
49	90	1.33E-22	1.96E-20	1.42E-18	6.73E-17	2.34E-15	6.38E-14
50	92	3.39E-23	5.12E-21	3.79E-19	1.83E-17	6.51E-16	1.81E-14
