# Supplementary material for: A Statistical Method for the Detection of Alternative Splicing Using RNA-Seq
Source: PLoS One. 2010 Jan 8;5(1):e8529. doi: 10.1371/journal.pone.0008529 (PMC2798953; doi:10.1371/journal.pone.0008529)
Supplement: Table S4 — Distribution of dinucleotide frequency across ESJ, rESJ and ERJ. (0.07 MB PDF) [file pone.0008529.s012.pdf]

Table S4. (A) ESJ dinucleotide

Position	AA	AC	AG	AT	Total	AA%	AC%	AG%	AT%
-21	152,631	113,302	146,696	112,780	525,409	29.05%	21.56%	27.92%	21.47%
-20	129,917	117,302	179,298	102,880	529,397	24.54%	22.16%	33.87%	19.43%
-19	123,651	109,950	157,674	93,918	485,193	25.48%	22.66%	32.50%	19.36%
-18	140,627	112,067	142,524	111,164	506,382	27.77%	22.13%	28.15%	21.95%
-17	131,486	119,245	172,945	100,740	524,416	25.07%	22.74%	32.98%	19.21%
-16	134,681	120,844	154,879	92,301	502,705	26.79%	24.04%	30.81%	18.36%
-15	149,923	116,931	149,819	113,974	530,647	28.25%	22.04%	28.23%	21.48%
-14	136,584	130,076	177,909	104,891	549,460	24.86%	23.67%	32.38%	19.09%
-13	127,549	117,582	151,350	94,593	491,074	25.97%	23.94%	30.82%	19.26%
-12	152,121	119,434	139,141	104,271	514,967	29.54%	23.19%	27.02%	20.25%
-11	147,713	125,240	178,569	100,887	552,409	26.74%	22.67%	32.33%	18.26%
-10	137,809	122,643	160,475	89,725	510,652	26.99%	24.02%	31.43%	17.57%
-9	149,203	113,988	135,573	125,539	524,303	28.46%	21.74%	25.86%	23.94%
-8	141,956	120,932	176,701	109,924	549,513	25.83%	22.01%	32.16%	20.00%
-7	140,231	111,364	149,454	101,785	502,834	27.89%	22.15%	29.72%	20.24%
-6	167,268	112,792	143,731	125,202	548,993	30.47%	20.55%	26.18%	22.81%
-5	181,667	134,931	161,774	108,584	586,956	30.95%	22.99%	27.56%	18.50%
-4	209,684	187,780	132,341	66,660	596,465	35.15%	31.48%	22.19%	11.18%
-3	432,272	70,485	85,113	83,338	671,208	64.40%	10.50%	12.68%	12.42%
-2	95,171	39,232	1,069,090	57,219	1,260,712	7.55%	3.11%	84.80%	4.54%
-1	53,403	29,852	95,649	25,758	204,662	26.09%	14.59%	46.74%	12.59%
1	130,284	84,099	106,987	191,366	512,736	25.41%	16.40%	20.87%	37.32%
2	150,594	106,214	105,760	115,679	478,247	31.49%	22.21%	22.11%	24.19%
3	137,146	123,826	111,126	132,091	504,189	27.20%	24.56%	22.04%	26.20%
4	150,021	108,399	101,822	123,472	483,714	31.01%	22.41%	21.05%	25.53%
5	151,765	121,581	153,222	107,482	534,050	28.42%	22.77%	28.69%	20.13%
6	141,241	104,276	162,793	96,392	504,702	27.99%	20.66%	32.26%	19.10%
7	159,925	103,035	142,149	119,816	524,925	30.47%	19.63%	27.08%	22.83%
8	151,917	119,181	187,338	124,430	582,866	26.06%	20.45%	32.14%	21.35%
9	133,827	102,286	167,735	95,570	499,418	26.80%	20.48%	33.59%	19.14%
10	162,462	102,780	144,388	114,538	524,168	30.99%	19.61%	27.55%	21.85%
11	147,431	113,075	170,827	108,691	540,024	27.30%	20.94%	31.63%	20.13%
12	134,693	100,199	162,854	101,899	499,645	26.96%	20.05%	32.59%	20.39%
13	151,672	101,515	136,890	114,577	504,654	30.05%	20.12%	27.13%	22.70%
14	149,970	122,162	170,173	106,022	548,327	27.35%	22.28%	31.03%	19.34%
15	135,253	102,469	152,488	96,342	486,552	27.80%	21.06%	31.34%	19.80%
16	151,403	102,870	148,469	112,878	515,620	29.36%	19.95%	28.79%	21.89%
17	138,062	112,673	177,164	105,964	533,863	25.86%	21.11%	33.19%	19.85%

18	134,667	98,993	160,665	90,996	485,321	27.75%	20.40%	33.10%	18.75%
19	157,466	102,044	142,570	107,115	509,195	30.92%	20.04%	28.00%	21.04%
20	145,640	111,907	180,333	111,784	549,664	26.50%	20.36%	32.81%	20.34%

**Position**	**CA**	**CC**	**CG**	**CT**	**Total**	**CA%**	**CC%**	**CG%**	**CT%**
-21	155,636	138,360	59,659	156,883	510,538	30.48%	27.10%	11.69%	30.73%
-20	158,381	139,414	61,780	150,964	510,539	31.02%	27.31%	12.10%	29.57%
-19	159,826	140,456	61,946	142,007	504,235	31.70%	27.86%	12.29%	28.16%
-18	157,879	132,205	59,390	155,415	504,889	31.27%	26.18%	11.76%	30.78%
-17	158,996	140,522	57,070	141,400	497,988	31.93%	28.22%	11.46%	28.39%
-16	167,345	140,538	60,379	147,496	515,758	32.45%	27.25%	11.71%	28.60%
-15	154,751	142,953	58,637	156,101	512,442	30.20%	27.90%	11.44%	30.46%
-14	159,304	147,587	54,784	147,233	508,908	31.30%	29.00%	10.77%	28.93%
-13	166,929	152,601	60,348	157,052	536,930	31.09%	28.42%	11.24%	29.25%
-12	173,656	138,358	61,543	156,785	530,342	32.74%	26.09%	11.60%	29.56%
-11	163,724	137,087	53,730	158,566	513,107	31.91%	26.72%	10.47%	30.90%
-10	166,383	139,396	56,500	142,771	505,050	32.94%	27.60%	11.19%	28.27%
-9	171,670	127,524	50,423	157,696	507,313	33.84%	25.14%	9.94%	31.08%
-8	151,135	130,906	52,804	142,705	477,550	31.65%	27.41%	11.06%	29.88%
-7	172,987	133,753	49,197	138,709	494,646	34.97%	27.04%	9.95%	28.04%
-6	162,788	120,102	41,626	153,945	478,461	34.02%	25.10%	8.70%	32.18%
-5	173,182	137,933	41,400	127,043	479,558	36.11%	28.76%	8.63%	26.49%
-4	210,335	187,980	43,043	79,018	520,376	40.42%	36.12%	8.27%	15.18%
-3	509,677	62,134	35,440	89,776	697,027	73.12%	8.91%	5.08%	12.88%
-2	55,751	18,302	98,819	48,952	221,824	25.13%	8.25%	44.55%	22.07%
-1	21,615	13,063	32,586	12,715	79,979	27.03%	16.33%	40.74%	15.90%
1	70,109	72,853	18,246	121,391	282,599	24.81%	25.78%	6.46%	42.96%
2	113,444	100,381	29,676	123,115	366,616	30.94%	27.38%	8.09%	33.58%
3	134,394	164,832	38,336	143,253	480,815	27.95%	34.28%	7.97%	29.79%
4	158,650	145,483	47,522	182,519	534,174	29.70%	27.24%	8.90%	34.17%
5	155,535	140,142	46,587	142,549	484,813	32.08%	28.91%	9.61%	29.40%
6	154,158	167,705	47,035	126,646	495,544	31.11%	33.84%	9.49%	25.56%
7	165,246	131,338	46,926	156,888	500,398	33.02%	26.25%	9.38%	31.35%
8	146,255	115,238	45,426	136,866	443,785	32.96%	25.97%	10.24%	30.84%
9	152,575	126,683	45,209	119,033	443,500	34.40%	28.56%	10.19%	26.84%
10	145,502	111,687	48,180	148,835	454,204	32.03%	24.59%	10.61%	32.77%
11	137,968	122,443	40,666	127,917	428,994	32.16%	28.54%	9.48%	29.82%
12	147,378	147,395	46,769	121,881	463,423	31.80%	31.81%	10.09%	26.30%
13	151,733	123,721	46,012	148,522	469,988	32.28%	26.32%	9.79%	31.60%
14	142,043	126,214	45,259	133,958	447,474	31.74%	28.21%	10.11%	29.94%

15	150,267	155,944	45,941	126,614	478,766	31.39%	32.57%	9.60%	26.45%
16	151,905	126,536	51,085	154,335	483,861	31.39%	26.15%	10.56%	31.90%
17	143,319	125,212	43,523	135,901	447,955	31.99%	27.95%	9.72%	30.34%
18	148,083	133,408	47,765	130,087	459,343	32.24%	29.04%	10.40%	28.32%
19	149,595	113,248	47,367	137,650	447,860	33.40%	25.29%	10.58%	30.74%
20	141,040	122,084	44,305	126,229	433,658	32.52%	28.15%	10.22%	29.11%

**Position**	**GA**	**GC**	**GG**	**GT**	**Total**	**GA%**	**GC%**	**GG%**	**GT%**
-21	162,922	139,219	124,337	97,707	524,185	31.08%	26.56%	23.72%	18.64%
-20	131,211	120,065	133,677	79,741	464,694	28.24%	25.84%	28.77%	17.16%
-19	157,587	142,378	149,698	97,215	546,878	28.82%	26.03%	27.37%	17.78%
-18	159,147	131,140	147,799	95,229	533,315	29.84%	24.59%	27.71%	17.86%
-17	142,344	126,605	129,201	83,540	481,690	29.55%	26.28%	26.82%	17.34%
-16	158,558	135,804	140,177	92,261	526,800	30.10%	25.78%	26.61%	17.51%
-15	172,460	129,576	117,898	85,683	505,617	34.11%	25.63%	23.32%	16.95%
-14	134,443	126,416	118,731	77,714	457,304	29.40%	27.64%	25.96%	16.99%
-13	151,737	139,871	128,094	92,356	512,058	29.63%	27.32%	25.02%	18.04%
-12	163,758	128,883	110,498	86,305	489,444	33.46%	26.33%	22.58%	17.63%
-11	133,395	115,454	117,660	83,544	450,053	29.64%	25.65%	26.14%	18.56%
-10	151,882	132,422	136,134	94,131	514,569	29.52%	25.73%	26.46%	18.29%
-9	160,339	126,275	136,714	99,560	522,888	30.66%	24.15%	26.15%	19.04%
-8	133,639	114,958	113,722	89,492	451,811	29.58%	25.44%	25.17%	19.81%
-7	158,384	126,508	119,926	112,935	517,753	30.59%	24.43%	23.16%	21.81%
-6	171,832	130,685	89,566	95,846	487,929	35.22%	26.78%	18.36%	19.64%
-5	140,990	114,377	82,500	66,617	404,484	34.86%	28.28%	20.40%	16.47%
-4	171,333	160,191	81,756	31,609	444,889	38.51%	36.01%	18.38%	7.10%
-3	250,965	41,877	39,182	43,007	375,031	66.92%	11.17%	10.45%	11.47%
-2	39,920	12,140	150,401	20,892	223,353	17.87%	5.44%	67.34%	9.35%
-1	399,268	217,685	754,230	171,571	1,542,754	25.88%	14.11%	48.89%	11.12%
1	247,111	168,313	197,893	338,874	952,191	25.95%	17.68%	20.78%	35.59%
2	114,549	100,573	87,523	104,491	407,136	28.14%	24.70%	21.50%	25.66%
3	134,431	103,983	127,001	102,299	467,714	28.74%	22.23%	27.15%	21.87%
4	149,777	100,163	94,098	98,987	443,025	33.81%	22.61%	21.24%	22.34%
5	115,703	95,393	103,590	79,526	394,212	29.35%	24.20%	26.28%	20.17%
6	161,736	112,076	131,026	94,949	499,787	32.36%	22.42%	26.22%	19.00%
7	182,621	109,270	106,884	106,548	505,323	36.14%	21.62%	21.15%	21.09%
8	129,410	94,467	107,807	89,736	421,420	30.71%	22.42%	25.58%	21.29%
9	167,646	112,972	152,036	96,070	528,724	31.71%	21.37%	28.76%	18.17%
10	175,356	115,879	147,776	106,929	545,940	32.12%	21.23%	27.07%	19.59%
11	144,586	112,057	116,226	99,421	472,290	30.61%	23.73%	24.61%	21.05%

12	162,299	115,973	136,960	94,192	509,424	31.86%	22.77%	26.89%	18.49%
13	183,224	119,448	110,246	106,224	519,142	35.29%	23.01%	21.24%	20.46%
14	127,934	104,593	111,702	83,527	427,756	29.91%	24.45%	26.11%	19.53%
15	166,531	116,665	130,266	97,079	510,541	32.62%	22.85%	25.52%	19.01%
16	170,614	113,916	109,451	96,635	490,616	34.78%	23.22%	22.31%	19.70%
17	137,731	108,195	115,761	87,779	449,466	30.64%	24.07%	25.76%	19.53%
18	164,060	112,021	148,316	97,120	521,517	31.46%	21.48%	28.44%	18.62%
19	180,135	113,360	139,054	96,923	529,472	34.02%	21.41%	26.26%	18.31%
20	143,502	110,475	122,256	91,055	467,288	30.71%	23.64%	26.16%	19.49%

**Position**	**TA**	**TC**	**TG**	**TT**	**Total**	**TA%**	**TC%**	**TG%**	**TT%**
-21	58,208	119,658	134,002	104,415	416,283	13.98%	28.74%	32.19%	25.08%
-20	65,684	127,454	172,123	106,524	471,785	13.92%	27.02%	36.48%	22.58%
-19	65,318	112,105	163,997	98,689	440,109	14.84%	25.47%	37.26%	22.42%
-18	66,763	122,576	131,977	110,513	431,829	15.46%	28.39%	30.56%	25.59%
-17	69,879	129,386	167,584	105,472	472,321	14.79%	27.39%	35.48%	22.33%
-16	70,063	115,256	150,182	95,651	431,152	16.25%	26.73%	34.83%	22.18%
-15	72,326	119,448	130,950	104,985	427,709	16.91%	27.93%	30.62%	24.55%
-14	60,743	132,851	160,634	106,515	460,743	13.18%	28.83%	34.86%	23.12%
-13	68,752	120,288	149,652	97,661	436,353	15.76%	27.57%	34.30%	22.38%
-12	62,874	126,432	138,871	113,485	441,662	14.24%	28.63%	31.44%	25.69%
-11	65,820	127,269	164,610	103,147	460,846	14.28%	27.62%	35.72%	22.38%
-10	68,229	112,852	169,779	95,284	446,144	15.29%	25.29%	38.05%	21.36%
-9	68,301	109,763	129,101	114,746	421,911	16.19%	26.02%	30.60%	27.20%
-8	76,104	127,850	174,526	119,061	497,541	15.30%	25.70%	35.08%	23.93%
-7	77,391	106,836	169,352	107,603	461,182	16.78%	23.17%	36.72%	23.33%
-6	85,068	115,979	129,561	130,424	461,032	18.45%	25.16%	28.10%	28.29%
-5	100,626	133,135	159,215	112,441	505,417	19.91%	26.34%	31.50%	22.25%
-4	79,856	161,076	117,891	55,862	414,685	19.26%	38.84%	28.43%	13.47%
-3	67,798	47,328	63,618	54,405	233,149	29.08%	20.30%	27.29%	23.33%
-2	13,820	10,305	224,444	21,957	270,526	5.11%	3.81%	82.97%	8.12%
-1	38,450	21,999	69,726	18,845	149,020	25.80%	14.76%	46.79%	12.65%
1	30,743	41,351	84,010	72,785	228,889	13.43%	18.07%	36.70%	31.80%
2	125,602	173,647	244,755	180,412	724,416	17.34%	23.97%	33.79%	24.90%
3	79,644	143,171	168,817	132,065	523,697	15.21%	27.34%	32.24%	25.22%
4	77,551	132,243	152,930	145,478	508,202	15.26%	26.02%	30.09%	28.63%
5	83,233	139,999	198,609	126,675	548,516	15.17%	25.52%	36.21%	23.09%
6	69,207	118,373	166,201	100,637	454,418	15.23%	26.05%	36.57%	22.15%
7	76,119	101,405	127,072	112,132	416,728	18.27%	24.33%	30.49%	26.91%
8	73,678	116,113	190,257	113,627	493,675	14.92%	23.52%	38.54%	23.02%

9	71,223	113,615	182,876	95,100	462,814	15.39%	24.55%	39.51%	20.55%
10	59,081	100,762	134,510	109,999	404,352	14.61%	24.92%	33.27%	27.20%
11	70,699	116,673	183,216	107,246	477,834	14.80%	24.42%	38.34%	22.44%
12	61,716	107,307	173,562	99,891	442,476	13.95%	24.25%	39.23%	22.58%
13	62,004	103,488	135,047	116,649	417,188	14.86%	24.81%	32.37%	27.96%
14	67,131	126,162	184,708	107,374	485,375	13.83%	25.99%	38.05%	22.12%
15	63,820	108,905	162,062	95,587	430,374	14.83%	25.30%	37.66%	22.21%
16	60,389	105,159	140,797	109,192	415,537	14.53%	25.31%	33.88%	26.28%
17	66,915	113,896	186,651	105,274	472,736	14.15%	24.09%	39.48%	22.27%
18	62,873	104,244	173,236	94,035	434,388	14.47%	24.00%	39.88%	21.65%
19	62,859	105,507	138,897	104,732	411,995	15.26%	25.61%	33.71%	25.42%
20	63,004	109,897	175,495	97,554	445,950	14.13%	24.64%	39.35%	21.88%

(B) ERJ dinucleotide

Position	AA	AC	AG	AT	Total	AA%	AC%	AG%	AT%
-21	194,244	110,703	142,591	132,939	580,477	33.46%	19.07%	24.56%	22.90%
-20	184,589	114,562	164,988	119,877	584,016	31.61%	19.62%	28.25%	20.53%
-19	181,602	106,150	150,692	114,387	552,831	32.85%	19.20%	27.26%	20.69%
-18	197,602	110,884	143,183	126,630	578,299	34.17%	19.17%	24.76%	21.90%
-17	183,958	117,318	166,839	116,264	584,379	31.48%	20.08%	28.55%	19.90%
-16	178,377	113,684	152,642	109,339	554,042	32.20%	20.52%	27.55%	19.73%
-15	187,604	116,336	148,079	119,884	571,903	32.80%	20.34%	25.89%	20.96%
-14	171,430	124,124	169,553	112,225	577,332	29.69%	21.50%	29.37%	19.44%
-13	156,725	113,580	152,435	106,430	529,170	29.62%	21.46%	28.81%	20.11%
-12	167,670	115,748	141,257	116,572	541,247	30.98%	21.39%	26.10%	21.54%
-11	155,971	119,274	164,104	114,862	554,211	28.14%	21.52%	29.61%	20.73%
-10	149,554	112,721	150,915	107,558	520,748	28.72%	21.65%	28.98%	20.65%
-9	162,133	112,342	133,767	129,006	537,248	30.18%	20.91%	24.90%	24.01%
-8	155,737	117,016	158,124	117,662	548,539	28.39%	21.33%	28.83%	21.45%
-7	164,581	107,285	140,400	113,005	525,271	31.33%	20.42%	26.73%	21.51%
-6	190,060	120,552	133,911	132,591	577,114	32.93%	20.89%	23.20%	22.97%
-5	187,875	131,746	154,942	114,866	589,429	31.87%	22.35%	26.29%	19.49%
-4	201,637	173,654	125,262	84,743	585,296	34.45%	29.67%	21.40%	14.48%
-3	378,778	75,698	86,350	93,903	634,729	59.68%	11.93%	13.60%	14.79%
-2	128,460	33,300	941,498	52,395	1,155,653	11.12%	2.88%	81.47%	4.53%
-1	70,305	38,200	122,257	32,261	263,023	26.73%	14.52%	46.48%	12.27%
1	122,817	89,179	125,866	187,796	525,658	23.36%	16.97%	23.94%	35.73%
2	147,287	98,970	113,241	110,510	470,008	31.34%	21.06%	24.09%	23.51%
3	138,053	118,876	120,144	121,674	498,747	27.68%	23.83%	24.09%	24.40%

4	142,498	108,667	113,663	116,962	481,790	29.58%	22.55%	23.59%	24.28%
5	146,220	108,688	152,919	107,352	515,179	28.38%	21.10%	29.68%	20.84%
6	140,148	103,699	165,002	95,546	504,395	27.79%	20.56%	32.71%	18.94%
7	151,828	103,611	146,012	112,488	513,939	29.54%	20.16%	28.41%	21.89%
8	144,060	110,818	176,182	113,274	544,334	26.47%	20.36%	32.37%	20.81%
9	135,345	101,380	162,492	97,167	496,384	27.27%	20.42%	32.74%	19.57%
10	153,277	104,615	151,401	111,354	520,647	29.44%	20.09%	29.08%	21.39%
11	143,109	109,097	174,135	107,101	533,442	26.83%	20.45%	32.64%	20.08%
12	138,068	101,840	161,263	98,404	499,575	27.64%	20.39%	32.28%	19.70%
13	152,975	104,433	148,440	111,811	517,659	29.55%	20.17%	28.68%	21.60%
14	139,040	114,315	169,965	107,094	530,414	26.21%	21.55%	32.04%	20.19%
15	131,486	104,315	154,242	96,173	486,216	27.04%	21.45%	31.72%	19.78%
16	145,348	105,285	150,539	109,200	510,372	28.48%	20.63%	29.50%	21.40%
17	134,714	109,609	172,452	103,821	520,596	25.88%	21.05%	33.13%	19.94%
18	133,828	100,192	158,550	90,868	483,438	27.68%	20.72%	32.80%	18.80%
19	152,265	106,942	151,648	107,582	518,437	29.37%	20.63%	29.25%	20.75%
20	141,544	111,161	177,030	106,880	536,615	26.38%	20.72%	32.99%	19.92%

**Position**	**CA**	**CC**	**CG**	**CT**	**Total**	**CA%**	**CC%**	**CG%**	**CT%**
-21	148,835	126,396	51,837	145,915	472,983	31.47%	26.72%	10.96%	30.85%
-20	150,572	129,206	52,985	136,118	468,881	32.11%	27.56%	11.30%	29.03%
-19	159,728	133,189	54,450	131,916	479,283	33.33%	27.79%	11.36%	27.52%
-18	148,432	123,605	53,056	143,964	469,057	31.64%	26.35%	11.31%	30.69%
-17	150,657	132,099	52,013	132,474	467,243	32.24%	28.27%	11.13%	28.35%
-16	162,990	132,695	55,033	140,761	491,479	33.16%	27.00%	11.20%	28.64%
-15	151,656	129,925	54,024	143,085	478,690	31.68%	27.14%	11.29%	29.89%
-14	154,283	136,732	51,220	141,619	483,854	31.89%	28.26%	10.59%	29.27%
-13	163,953	138,555	54,879	149,456	506,843	32.35%	27.34%	10.83%	29.49%
-12	157,597	131,358	53,488	154,482	496,925	31.71%	26.43%	10.76%	31.09%
-11	160,029	135,558	49,370	149,063	494,020	32.39%	27.44%	9.99%	30.17%
-10	164,563	130,595	52,121	147,462	494,741	33.26%	26.40%	10.54%	29.81%
-9	154,028	123,229	46,824	153,449	477,530	32.26%	25.81%	9.81%	32.13%
-8	154,002	127,298	46,880	145,097	473,277	32.54%	26.90%	9.91%	30.66%
-7	168,230	125,689	45,586	143,375	482,880	34.84%	26.03%	9.44%	29.69%
-6	152,886	119,985	38,376	148,574	459,821	33.25%	26.09%	8.35%	32.31%
-5	165,335	138,118	38,090	135,610	477,153	34.65%	28.95%	7.98%	28.42%
-4	197,000	186,182	38,967	95,942	518,091	38.02%	35.94%	7.52%	18.52%
-3	465,452	66,478	34,323	98,948	665,201	69.97%	9.99%	5.16%	14.87%
-2	60,920	37,490	91,790	54,562	244,762	24.89%	15.32%	37.50%	22.29%
-1	34,321	19,090	59,429	16,025	128,865	26.63%	14.81%	46.12%	12.44%

1	69,703	75,213	27,879	115,493	288,288	24.18%	26.09%	9.67%	40.06%
2	113,682	103,345	40,609	126,476	384,112	29.60%	26.90%	10.57%	32.93%
3	134,011	141,433	48,026	145,712	469,182	28.56%	30.14%	10.24%	31.06%
4	149,002	143,599	56,355	167,882	516,838	28.83%	27.78%	10.90%	32.48%
5	156,073	140,889	55,015	148,907	500,884	31.16%	28.13%	10.98%	29.73%
6	151,045	142,295	58,974	136,162	488,476	30.92%	29.13%	12.07%	27.87%
7	150,191	131,818	57,652	147,917	487,578	30.80%	27.04%	11.82%	30.34%
8	147,071	129,478	56,620	140,557	473,726	31.05%	27.33%	11.95%	29.67%
9	150,879	136,256	56,540	131,013	474,688	31.78%	28.70%	11.91%	27.60%
10	147,581	124,665	58,139	148,085	478,470	30.84%	26.05%	12.15%	30.95%
11	143,592	131,836	55,521	134,098	465,047	30.88%	28.35%	11.94%	28.84%
12	147,894	137,323	58,690	133,237	477,144	31.00%	28.78%	12.30%	27.92%
13	144,848	129,848	56,805	147,864	479,365	30.22%	27.09%	11.85%	30.85%
14	144,196	135,876	57,045	138,429	475,546	30.32%	28.57%	12.00%	29.11%
15	152,140	146,472	59,875	138,217	496,704	30.63%	29.49%	12.05%	27.83%
16	148,160	134,170	61,049	152,876	496,255	29.86%	27.04%	12.30%	30.81%
17	143,456	135,160	57,971	141,651	478,238	30.00%	28.26%	12.12%	29.62%
18	153,060	138,436	59,850	135,118	486,464	31.46%	28.46%	12.30%	27.78%
19	149,995	127,313	58,884	142,585	478,777	31.33%	26.59%	12.30%	29.78%
20	145,583	134,777	56,843	135,098	472,301	30.82%	28.54%	12.04%	28.60%

**Position**	**GA**	**GC**	**GG**	**GT**	**Total**	**GA%**	**GC%**	**GG%**	**GT%**
-21	157,627	122,450	117,760	91,169	489,006	32.23%	25.04%	24.08%	18.64%
-20	129,791	113,577	119,540	78,598	441,506	29.40%	25.72%	27.08%	17.80%
-19	150,521	122,556	136,358	89,203	498,638	30.19%	24.58%	27.35%	17.89%
-18	154,421	122,586	123,919	90,257	491,183	31.44%	24.96%	25.23%	18.38%
-17	134,359	118,764	115,538	80,000	448,661	29.95%	26.47%	25.75%	17.83%
-16	151,543	122,378	131,270	87,598	492,789	30.75%	24.83%	26.64%	17.78%
-15	161,890	121,601	113,457	88,951	485,899	33.32%	25.03%	23.35%	18.31%
-14	132,074	117,891	116,016	80,417	446,398	29.59%	26.41%	25.99%	18.01%
-13	147,728	127,708	125,112	90,106	490,654	30.11%	26.03%	25.50%	18.36%
-12	156,572	123,518	108,370	89,626	478,086	32.75%	25.84%	22.67%	18.75%
-11	128,756	111,893	112,940	84,104	437,693	29.42%	25.56%	25.80%	19.22%
-10	144,201	120,412	127,428	91,258	483,299	29.84%	24.91%	26.37%	18.88%
-9	153,088	119,018	116,104	97,224	485,434	31.54%	24.52%	23.92%	20.03%
-8	128,421	108,526	107,574	86,222	430,743	29.81%	25.20%	24.97%	20.02%
-7	154,430	113,469	114,102	94,845	476,846	32.39%	23.80%	23.93%	19.89%
-6	157,759	118,234	89,450	94,522	459,965	34.30%	25.70%	19.45%	20.55%
-5	132,816	110,474	82,299	66,278	391,867	33.89%	28.19%	21.00%	16.91%
-4	154,022	147,547	82,203	44,873	428,645	35.93%	34.42%	19.18%	10.47%

-3	224,196	46,199	43,381	52,552	366,328	61.20%	12.61%	11.84%	14.35%
-2	48,465	25,998	147,535	31,751	253,749	19.10%	10.25%	58.14%	12.51%
-1	372,649	204,281	655,275	170,046	1,402,251	26.58%	14.57%	46.73%	12.13%
1	242,670	175,396	189,172	314,650	921,888	26.32%	19.03%	20.52%	34.13%
2	123,509	105,417	97,267	103,026	429,219	28.78%	24.56%	22.66%	24.00%
3	137,017	114,883	137,106	100,319	489,325	28.00%	23.48%	28.02%	20.50%
4	149,772	115,101	104,178	100,426	469,477	31.90%	24.52%	22.19%	21.39%
5	123,624	107,618	112,354	85,557	429,153	28.81%	25.08%	26.18%	19.94%
6	154,388	120,882	138,779	96,292	510,341	30.25%	23.69%	27.19%	18.87%
7	176,450	125,066	122,969	108,487	532,972	33.11%	23.47%	23.07%	20.36%
8	136,417	113,559	122,709	94,461	467,146	29.20%	24.31%	26.27%	20.22%
9	164,249	123,811	147,855	98,408	534,323	30.74%	23.17%	27.67%	18.42%
10	169,827	127,294	138,141	104,523	539,785	31.46%	23.58%	25.59%	19.36%
11	144,324	119,541	133,793	96,575	494,233	29.20%	24.19%	27.07%	19.54%
12	167,163	129,338	145,977	101,403	543,881	30.74%	23.78%	26.84%	18.64%
13	169,474	131,600	128,807	104,764	534,645	31.70%	24.61%	24.09%	19.60%
14	137,379	122,389	129,513	90,980	480,261	28.61%	25.48%	26.97%	18.94%
15	161,688	131,431	141,940	99,443	534,502	30.25%	24.59%	26.56%	18.60%
16	164,316	127,848	129,143	100,353	521,660	31.50%	24.51%	24.76%	19.24%
17	139,515	123,948	132,744	92,698	488,905	28.54%	25.35%	27.15%	18.96%
18	165,846	128,544	150,935	97,494	542,819	30.55%	23.68%	27.81%	17.96%
19	174,134	127,206	136,822	100,235	538,397	32.34%	23.63%	25.41%	18.62%
20	143,980	121,429	133,208	91,918	490,535	29.35%	24.75%	27.16%	18.74%

Position	TA	TC	TG	TT	Total	TA%	TC%	TG%	TT%
-21	83,310	109,332	129,318	111,985	433,945	19.20%	25.19%	29.80%	25.81%
-20	87,879	121,938	161,125	111,066	482,008	18.23%	25.30%	33.43%	23.04%
-19	86,448	107,162	149,683	102,366	445,659	19.40%	24.05%	33.59%	22.97%
-18	83,924	110,168	128,503	115,277	437,872	19.17%	25.16%	29.35%	26.33%
-17	85,068	123,298	158,399	109,363	476,128	17.87%	25.90%	33.27%	22.97%
-16	78,993	109,933	146,954	102,221	438,101	18.03%	25.09%	33.54%	23.33%
-15	76,182	115,992	130,838	116,907	439,919	17.32%	26.37%	29.74%	26.57%
-14	71,383	128,096	153,865	115,483	468,827	15.23%	27.32%	32.82%	24.63%
-13	72,841	117,082	145,660	114,161	449,744	16.20%	26.03%	32.39%	25.38%
-12	72,372	123,396	134,578	129,807	460,153	15.73%	26.82%	29.25%	28.21%
-11	75,992	128,016	156,885	129,594	490,487	15.49%	26.10%	31.99%	26.42%
-10	78,930	113,802	154,970	129,921	477,623	16.53%	23.83%	32.45%	27.20%
-9	79,290	118,688	134,048	144,173	476,199	16.65%	24.92%	28.15%	30.28%
-8	87,111	130,040	164,268	142,433	523,852	16.63%	24.82%	31.36%	27.19%
-7	89,873	113,378	159,877	128,286	491,414	18.29%	23.07%	32.53%	26.11%

-6	88,724	118,382	130,130	142,275	479,511	18.50%	24.69%	27.14%	29.67%
-5	99,270	137,753	153,314	127,625	517,962	19.17%	26.60%	29.60%	24.64%
-4	82,070	157,818	119,896	84,595	444,379	18.47%	35.51%	26.98%	19.04%
-3	87,227	56,387	89,695	76,844	310,153	28.12%	18.18%	28.92%	24.78%
-2	25,178	32,077	221,428	43,564	322,247	7.81%	9.95%	68.71%	13.52%
-1	48,383	26,717	84,927	22,245	182,272	26.54%	14.66%	46.59%	12.20%
1	34,818	44,324	86,302	75,133	240,577	14.47%	18.42%	35.87%	31.23%
2	114,269	161,450	238,208	179,145	693,072	16.49%	23.29%	34.37%	25.85%
3	72,864	141,817	164,392	140,084	519,157	14.04%	27.32%	31.67%	26.98%
4	74,093	133,696	155,206	144,667	507,662	14.59%	26.34%	30.57%	28.50%
5	78,652	131,448	190,284	129,368	529,752	14.85%	24.81%	35.92%	24.42%
6	68,502	120,884	170,420	111,171	470,977	14.54%	25.67%	36.18%	23.60%
7	66,015	113,436	140,766	118,795	439,012	15.04%	25.84%	32.06%	27.06%
8	69,153	121,053	179,296	117,960	487,462	14.19%	24.83%	36.78%	24.20%
9	70,367	117,188	173,219	105,243	466,017	15.10%	25.15%	37.17%	22.58%
10	63,060	108,715	147,113	112,763	431,651	14.61%	25.19%	34.08%	26.12%
11	68,893	116,985	181,325	109,186	476,389	14.46%	24.56%	38.06%	22.92%
12	64,803	111,059	169,097	101,715	446,674	14.51%	24.86%	37.86%	22.77%
13	63,238	109,789	146,537	115,029	434,593	14.55%	25.26%	33.72%	26.47%
14	65,929	124,353	178,918	110,115	479,315	13.75%	25.94%	37.33%	22.97%
15	65,231	114,134	165,935	101,047	446,347	14.61%	25.57%	37.18%	22.64%
16	62,984	111,100	148,688	112,021	434,793	14.49%	25.55%	34.20%	25.76%
17	66,189	118,119	181,068	108,916	474,292	13.96%	24.90%	38.18%	22.96%
18	65,924	111,815	169,737	99,176	446,652	14.76%	25.03%	38.00%	22.20%
19	60,433	111,061	144,032	106,953	422,479	14.30%	26.29%	34.09%	25.32%
20	65,154	115,830	174,563	101,588	457,135	14.25%	25.34%	38.19%	22.22%

(C) rESJ dinucleotide

Position	AA	AC	AG	AT	Total	AA%	AC%	AG%	AT%
-21	207,644	114,917	145,084	137,437	605,082	34.32%	18.99%	23.98%	22.71%
-20	192,444	119,067	180,737	125,740	617,988	31.14%	19.27%	29.25%	20.35%
-19	184,257	112,562	159,016	118,739	574,574	32.07%	19.59%	27.68%	20.67%
-18	203,718	115,350	144,365	135,537	598,970	34.01%	19.26%	24.10%	22.63%
-17	184,357	122,121	176,167	121,280	603,925	30.53%	20.22%	29.17%	20.08%
-16	179,499	122,942	158,821	113,322	574,584	31.24%	21.40%	27.64%	19.72%
-15	187,298	119,905	149,133	132,604	588,940	31.80%	20.36%	25.32%	22.52%
-14	173,562	132,340	179,711	115,882	601,495	28.86%	22.00%	29.88%	19.27%
-13	161,764	122,364	157,052	108,528	549,708	29.43%	22.26%	28.57%	19.74%
-12	182,537	120,286	140,013	121,878	564,714	32.32%	21.30%	24.79%	21.58%

-11	161,020	130,527	182,133	123,447	597,127	26.97%	21.86%	30.50%	20.67%
-10	157,206	115,656	161,512	106,884	541,258	29.04%	21.37%	29.84%	19.75%
-9	171,663	118,537	137,036	143,870	571,106	30.06%	20.76%	23.99%	25.19%
-8	160,963	123,243	170,739	121,824	576,769	27.91%	21.37%	29.60%	21.12%
-7	166,876	113,741	145,521	116,039	542,177	30.78%	20.98%	26.84%	21.40%
-6	194,208	121,879	141,977	137,571	595,635	32.61%	20.46%	23.84%	23.10%
-5	199,497	142,559	157,887	120,587	620,530	32.15%	22.97%	25.44%	19.43%
-4	212,083	184,198	131,090	87,172	614,543	34.51%	29.97%	21.33%	14.18%
-3	411,492	75,081	88,741	94,009	669,323	61.48%	11.22%	13.26%	14.05%
-2	138,668	39,844	981,116	58,618	1,218,246	11.38%	3.27%	80.54%	4.81%
-1	75,838	44,208	133,781	34,154	287,981	26.33%	15.35%	46.45%	11.86%
1	135,985	88,602	133,844	187,484	545,915	24.91%	16.23%	24.52%	34.34%
2	145,804	107,648	112,666	116,715	482,833	30.20%	22.30%	23.33%	24.17%
3	137,231	118,992	122,270	124,965	503,458	27.26%	23.63%	24.29%	24.82%
4	145,084	106,301	114,353	119,811	485,549	29.88%	21.89%	23.55%	24.68%
5	146,876	116,704	156,447	105,828	525,855	27.93%	22.19%	29.75%	20.12%
6	139,082	103,536	161,932	96,926	501,476	27.73%	20.65%	32.29%	19.33%
7	155,890	102,527	147,956	115,796	522,169	29.85%	19.63%	28.33%	22.18%
8	147,259	115,934	181,352	119,057	563,602	26.13%	20.57%	32.18%	21.12%
9	134,227	98,466	169,205	97,766	499,664	26.86%	19.71%	33.86%	19.57%
10	160,164	101,188	150,948	109,965	522,265	30.67%	19.37%	28.90%	21.06%
11	144,391	107,370	172,375	109,260	533,396	27.07%	20.13%	32.32%	20.48%
12	132,749	100,270	163,831	100,057	496,907	26.72%	20.18%	32.97%	20.14%
13	146,756	100,536	147,683	108,846	503,821	29.13%	19.95%	29.31%	21.60%
14	145,589	112,001	168,970	106,827	533,387	27.30%	21.00%	31.68%	20.03%
15	134,575	104,172	156,699	96,691	492,137	27.35%	21.17%	31.84%	19.65%
16	142,224	99,469	153,235	107,786	502,714	28.29%	19.79%	30.48%	21.44%
17	135,183	105,609	177,054	104,879	522,725	25.86%	20.20%	33.87%	20.06%
18	137,806	96,867	164,840	90,974	490,487	28.10%	19.75%	33.61%	18.55%
19	155,367	101,407	149,473	105,595	511,842	30.35%	19.81%	29.20%	20.63%
20	145,549	109,762	175,997	110,589	541,897	26.86%	20.26%	32.48%	20.41%

**Position**	**CA**	**CC**	**CG**	**CT**	**Total**	**CA%**	**CC%**	**CG%**	**CT%**
-21	159,923	133,641	46,318	147,544	487,426	32.81%	27.42%	9.50%	30.27%
-20	165,124	131,360	45,131	149,495	491,110	33.62%	26.75%	9.19%	30.44%
-19	166,479	134,145	46,967	133,734	481,325	34.59%	27.87%	9.76%	27.78%
-18	156,583	120,655	46,041	153,787	477,066	32.82%	25.29%	9.65%	32.24%
-17	161,138	129,794	43,724	131,540	466,196	34.56%	27.84%	9.38%	28.22%
-16	172,627	136,217	48,434	136,755	494,033	34.94%	27.57%	9.80%	27.68%
-15	159,471	138,138	44,975	158,415	500,999	31.83%	27.57%	8.98%	31.62%

-14	167,356	138,890	41,337	147,041	494,624	33.83%	28.08%	8.36%	29.73%
-13	172,369	145,838	48,023	153,686	519,916	33.15%	28.05%	9.24%	29.56%
-12	171,943	133,867	46,122	161,519	513,451	33.49%	26.07%	8.98%	31.46%
-11	171,371	132,178	40,682	153,698	497,929	34.42%	26.55%	8.17%	30.87%
-10	174,463	136,059	44,053	143,482	498,057	35.03%	27.32%	8.84%	28.81%
-9	168,724	121,532	37,844	155,267	483,367	34.91%	25.14%	7.83%	32.12%
-8	159,016	128,761	38,222	145,791	471,790	33.70%	27.29%	8.10%	30.90%
-7	180,281	138,299	37,557	142,790	498,927	36.13%	27.72%	7.53%	28.62%
-6	165,656	127,129	30,854	158,211	481,850	34.38%	26.38%	6.40%	32.83%
-5	178,364	141,244	29,939	140,385	489,932	36.41%	28.83%	6.11%	28.65%
-4	211,746	192,580	33,346	98,046	535,718	39.53%	35.95%	6.22%	18.30%
-3	486,293	67,835	28,889	99,108	682,125	71.29%	9.94%	4.24%	14.53%
-2	71,872	35,468	85,320	57,247	249,907	28.76%	14.19%	34.14%	22.91%
-1	36,482	21,041	52,307	19,393	129,223	28.23%	16.28%	40.48%	15.01%
1	75,183	78,419	29,235	123,299	306,136	24.56%	25.62%	9.55%	40.28%
2	115,088	103,404	42,707	126,066	387,265	29.72%	26.70%	11.03%	32.55%
3	135,832	160,743	51,286	144,580	492,441	27.58%	32.64%	10.41%	29.36%
4	156,036	144,038	57,979	180,254	538,307	28.99%	26.76%	10.77%	33.49%
5	152,831	143,931	58,337	147,597	502,696	30.40%	28.63%	11.60%	29.36%
6	154,446	160,279	60,595	136,541	511,861	30.17%	31.31%	11.84%	26.68%
7	156,786	138,673	59,826	154,567	509,852	30.75%	27.20%	11.73%	30.32%
8	147,608	124,756	60,366	144,448	477,178	30.93%	26.14%	12.65%	30.27%
9	152,675	130,629	57,849	125,029	466,182	32.75%	28.02%	12.41%	26.82%
10	143,248	122,749	60,235	147,647	473,879	30.23%	25.90%	12.71%	31.16%
11	136,264	128,783	55,048	135,572	455,667	29.90%	28.26%	12.08%	29.75%
12	144,410	144,601	60,097	126,040	475,148	30.39%	30.43%	12.65%	26.53%
13	148,319	129,155	59,476	149,993	486,943	30.46%	26.52%	12.21%	30.80%
14	144,891	130,529	57,687	139,886	472,993	30.63%	27.60%	12.20%	29.57%
15	144,580	146,786	60,961	132,515	484,842	29.82%	30.28%	12.57%	27.33%
16	148,209	133,610	63,745	151,638	497,202	29.81%	26.87%	12.82%	30.50%
17	143,713	129,652	56,777	139,931	470,073	30.57%	27.58%	12.08%	29.77%
18	145,532	132,378	60,324	133,074	471,308	30.88%	28.09%	12.80%	28.24%
19	145,629	121,029	60,507	139,667	466,832	31.20%	25.93%	12.96%	29.92%
20	137,295	126,532	55,843	132,875	452,545	30.34%	27.96%	12.34%	29.36%

Position	GA	GC	GG	GT	Total	GA%	GC%	GG%	GT%
-21	165,027	125,918	110,644	96,415	498,004	33.14%	25.28%	22.22%	19.36%
-20	126,364	105,239	120,628	78,112	430,343	29.36%	24.45%	28.03%	18.15%
-19	156,913	121,104	141,333	94,848	514,198	30.52%	23.55%	27.49%	18.45%
-18	153,444	120,213	141,894	94,001	509,552	30.11%	23.59%	27.85%	18.45%

-17	137,837	115,104	120,787	84,771	458,499	30.06%	25.10%	26.34%	18.49%
-16	154,407	129,813	132,869	95,664	512,753	30.11%	25.32%	25.91%	18.66%
-15	173,854	118,672	111,368	90,083	493,977	35.19%	24.02%	22.55%	18.24%
-14	131,586	112,631	109,259	82,868	436,344	30.16%	25.81%	25.04%	18.99%
-13	154,061	123,420	123,484	98,808	499,773	30.83%	24.70%	24.71%	19.77%
-12	166,072	118,159	103,639	95,352	483,222	34.37%	24.45%	21.45%	19.73%
-11	129,645	105,112	106,913	86,689	428,359	30.27%	24.54%	24.96%	20.24%
-10	157,466	116,961	128,193	95,254	497,874	31.63%	23.49%	25.75%	19.13%
-9	155,651	117,063	130,258	100,531	503,503	30.91%	23.25%	25.87%	19.97%
-8	133,530	109,529	105,015	92,325	440,399	30.32%	24.87%	23.85%	20.96%
-7	158,662	114,251	110,582	105,456	488,951	32.45%	23.37%	22.62%	21.57%
-6	169,058	119,609	81,165	98,110	467,942	36.13%	25.56%	17.35%	20.97%
-5	130,748	110,985	71,963	69,029	382,725	34.16%	29.00%	18.80%	18.04%
-4	159,280	142,838	77,316	41,292	420,726	37.86%	33.95%	18.38%	9.81%
-3	232,452	45,813	39,846	54,185	372,296	62.44%	12.31%	10.70%	14.55%
-2	49,573	23,773	134,251	30,171	237,768	20.85%	10.00%	56.46%	12.69%
-1	383,690	212,448	670,148	163,980	1,430,266	26.83%	14.85%	46.85%	11.47%
1	237,935	176,077	207,500	319,136	940,648	25.29%	18.72%	22.06%	33.93%
2	126,034	115,635	101,744	111,954	455,367	27.68%	25.39%	22.34%	24.59%
3	139,543	113,917	140,154	104,790	498,404	28.00%	22.86%	28.12%	21.03%
4	152,613	118,985	106,767	108,520	486,885	31.34%	24.44%	21.93%	22.29%
5	120,382	112,494	111,096	87,987	431,959	27.87%	26.04%	25.72%	20.37%
6	161,217	125,302	139,423	98,081	524,023	30.77%	23.91%	26.61%	18.72%
7	175,857	127,411	118,548	111,972	533,788	32.95%	23.87%	22.21%	20.98%
8	132,675	109,990	124,997	93,239	460,901	28.79%	23.86%	27.12%	20.23%
9	163,683	127,379	154,466	103,567	549,095	29.81%	23.20%	28.13%	18.86%
10	170,802	128,877	156,020	109,037	564,736	30.24%	22.82%	27.63%	19.31%
11	148,043	124,321	134,040	102,171	508,575	29.11%	24.44%	26.36%	20.09%
12	164,817	130,065	148,615	101,259	544,756	30.26%	23.88%	27.28%	18.59%
13	176,114	133,354	126,446	106,846	542,760	32.45%	24.57%	23.30%	19.69%
14	134,293	120,496	127,187	91,042	473,018	28.39%	25.47%	26.89%	19.25%
15	158,982	131,417	142,665	103,079	536,143	29.65%	24.51%	26.61%	19.23%
16	170,934	127,726	128,904	100,436	528,000	32.37%	24.19%	24.41%	19.02%
17	144,126	119,014	136,533	91,418	491,091	29.35%	24.23%	27.80%	18.62%
18	166,513	129,370	155,863	100,141	551,887	30.17%	23.44%	28.24%	18.15%
19	177,720	122,422	155,224	99,183	554,549	32.05%	22.08%	27.99%	17.89%
20	153,378	123,728	135,750	93,844	506,700	30.27%	24.42%	26.79%	18.52%

**Position**	**TA**	**TC**	**TG**	**TT**	**Total**	**TA%**	**TC%**	**TG%**	**TT%**
-21	85,394	116,634	128,297	112,073	442,398	19.30%	26.36%	29.00%	25.33%

-20	90,642	125,659	167,702	109,466	493,469	18.37%	25.46%	33.98%	22.18%
-19	91,321	109,255	162,236	100,001	462,813	19.73%	23.61%	35.05%	21.61%
-18	90,180	109,978	126,199	120,965	447,322	20.16%	24.59%	28.21%	27.04%
-17	91,252	127,014	172,075	113,949	504,290	18.10%	25.19%	34.12%	22.60%
-16	82,407	112,027	153,853	103,253	451,540	18.25%	24.81%	34.07%	22.87%
-15	80,872	117,909	130,868	119,345	448,994	18.01%	26.26%	29.15%	26.58%
-14	77,204	136,055	169,466	117,722	500,447	15.43%	27.19%	33.86%	23.52%
-13	76,520	121,829	154,663	110,501	463,513	16.51%	26.28%	33.37%	23.84%
-12	76,575	125,617	138,585	130,746	471,523	16.24%	26.64%	29.39%	27.73%
-11	79,222	130,240	168,146	131,887	509,495	15.55%	25.56%	33.00%	25.89%
-10	81,971	114,691	169,745	129,314	495,721	16.54%	23.14%	34.24%	26.09%
-9	80,731	114,658	135,261	144,284	474,934	17.00%	24.14%	28.48%	30.38%
-8	88,668	137,394	174,975	142,915	543,952	16.30%	25.26%	32.17%	26.27%
-7	89,816	115,559	174,282	123,198	502,855	17.86%	22.98%	34.66%	24.50%
-6	91,608	121,315	128,729	145,831	487,483	18.79%	24.89%	26.41%	29.92%
-5	105,934	140,930	160,937	131,922	539,723	19.63%	26.11%	29.82%	24.44%
-4	86,214	162,509	130,544	82,656	461,923	18.66%	35.18%	28.26%	17.89%
-3	88,009	61,178	80,292	79,687	309,166	28.47%	19.79%	25.97%	25.77%
-2	27,868	30,138	229,579	39,404	326,989	8.52%	9.22%	70.21%	12.05%
-1	49,905	28,439	84,412	22,684	185,440	26.91%	15.34%	45.52%	12.23%
1	33,730	44,167	84,788	77,526	240,211	14.04%	18.39%	35.30%	32.27%
2	116,532	165,754	241,287	183,872	707,445	16.47%	23.43%	34.11%	25.99%
3	74,188	146,836	174,804	142,779	538,607	13.77%	27.26%	32.45%	26.51%
4	73,543	135,532	154,438	151,370	514,883	14.28%	26.32%	29.99%	29.40%
5	82,495	140,962	199,514	134,654	557,625	14.79%	25.28%	35.78%	24.15%
6	68,585	122,929	173,285	108,867	473,666	14.48%	25.95%	36.58%	22.98%
7	75,992	110,310	135,628	116,240	438,170	17.34%	25.18%	30.95%	26.53%
8	73,420	117,510	184,213	121,552	496,695	14.78%	23.66%	37.09%	24.47%
9	72,636	119,248	184,493	99,812	476,189	15.25%	25.04%	38.74%	20.96%
10	60,973	105,439	143,333	114,769	424,514	14.36%	24.84%	33.76%	27.04%
11	68,968	115,699	184,658	108,967	478,292	14.42%	24.19%	38.61%	22.78%
12	62,900	112,983	170,980	108,096	454,959	13.83%	24.83%	37.58%	23.76%
13	62,558	110,543	139,837	121,306	434,244	14.41%	25.46%	32.20%	27.93%
14	67,750	122,219	183,750	112,614	486,333	13.93%	25.13%	37.78%	23.16%
15	64,795	114,906	167,892	102,308	449,901	14.40%	25.54%	37.32%	22.74%
16	61,703	109,596	145,820	117,380	434,499	14.20%	25.22%	33.56%	27.02%
17	67,985	117,561	183,336	108,015	476,897	14.26%	24.65%	38.44%	22.65%
18	62,304	108,767	174,381	98,195	443,647	14.04%	24.52%	39.31%	22.13%
19	63,434	108,152	142,307	108,149	422,042	15.03%	25.63%	33.72%	25.63%
20	64,931	115,578	169,591	102,042	452,142	14.36%	25.56%	37.51%	22.57%
